# Using affinity propagation clustering for identifying bacterial clades and subclades with whole-genome sequences of *Francisella tularensis*

**DOI:** 10.1371/journal.pntd.0008018

**Published:** 2020-09-29

**Authors:** Anne Busch, Timo Homeier-Bachmann, Mostafa Y. Abdel-Glil, Anja Hackbart, Helmut Hotzel, Herbert Tomaso

**Affiliations:** 1 Friedrich-Loeffler-Institut, Federal Research Institute for Animal Health, Institute of Bacterial Infections and Zoonoses, Friedrich-Loeffler-Institut, Jena, Germany; 2 Friedrich-Loeffler-Institut, Federal Research Institute for Animal Health, Institute of Epidemiology, Friedrich-Loeffler-Institut, Greifswald-Insel Riems, Germany; Beijing Institute of Microbiology and Epidemiology, CHINA

## Abstract

By combining a reference-independent SNP analysis and average nucleotide identity (ANI) with affinity propagation clustering (APC), we developed a significantly improved methodology allowing resolving phylogenetic relationships, based on objective criteria. These bioinformatics tools can be used as a general ruler to determine phylogenetic relationships and clustering of bacteria, exemplary done with *Francisella* (*F*.) *tularensis*. Molecular epidemiology of *F*. *tularensis* is currently assessed mostly based on laboratory methods and molecular analysis. The high evolutionary stability and the clonal nature makes *Francisella* ideal for subtyping with single nucleotide polymorphisms (SNPs). Sequencing and real-time PCR can be used to validate the SNP analysis. We investigate whole-genome sequences of 155 *F*. *tularensis* subsp. *holarctica* isolates. Phylogenetic testing was based on SNPs and average nucleotide identity (ANI) as reference independent, alignment-free methods taking small-scale and large-scale differences within the genomes into account. Especially the whole genome SNP analysis with kSNP3.0 allowed deciphering quite subtle signals of systematic differences in molecular variation. Affinity propagation clustering (APC) resulted in three clusters showing the known clades B.4, B.6, and B.12. These data correlated with the results of real‐time PCR assays targeting canSNPs loci. Additionally, we detected two subtle sub-clusters. SplitsTree was used with standard-setting using the aligned SNPs from Parsnps. Together APC, HierBAPS, and SplitsTree enabled us to generate hypotheses about epidemiologic relationships between bacterial clusters and describing the distribution of isolates. Our data indicate that the choice of the typing technique can increase our understanding of the pathogenesis and transmission of diseases with the eventual for prevention. This is opening perspectives to be applied to other bacterial species. The data provide evidence that Germany might be the collision zone where the clade B.12, also known as the East European clade, overlaps with the clade B.6, also known as the Iberian clade. Described methods allow generating a new, more detailed perspective for *F*. *tularensis* subsp. *holarctica* phylogeny. These results may encourage to determine phylogenetic relationships and clustering of other bacteria the same way.

## Author summary

By combining a reference-independent SNP analysis and ANI (average nucleotide identity) with affinity propagation clustering (APC), we tested a methodology allowing resolving phylogenetic relationships, based on objective criteria. These bioinformatics tools can be used as a general ruler to determine phylogenetic relationships and clustering of bacteria, exemplary done with *Francisella tularensis*. *Francisella tularensis* causes the zoonosis tularemia. We analyzed the relationships between *Francisella tularensis* subsp. *holarctica* isolates from Germany using whole-genome sequences. We chose open source, reference independent methods to optimize the level of discrimination. Using a recently described clustering algorithm, we exploit a novel approach to the clustering of bacteria. APC can be used for assigning clades and can be used for rapidly typing strains when they arise. Additionally, we detected two sub-clusters. The data provide evidence that Germany is the collision zone where the clade B.12, also known as the East European clade, overlaps with the clade B.6, also known as the Iberian clade.

## Introduction

*Francisella* (*F*.) *tularensis* is the causative agent of tularemia and a highly infectious, Gram-negative, bacterial pathogen [1–3]. *F*. *tularensis* is listed as a category A bioterrorism agent, not only due to its very low infectious dose but also due to its pathogenicity and its virulence, the ability to multiply within host cells and its ability of intracellular survival [4, 5]. Tularemia is a febrile disease that may be severe to fatal. Prompt antibiotic treatment avoids severe complications [6]. The major causes of tularemia in humans are the two subspecies *F*. *tularensis* subsp. *tularensis* and *F*. *tularensis* subsp. *holarctica* [7]. Only the less pathogenic *F*. *tularensis* subsp. *holarctica* is endemic in Europe [2, 8]. In Germany and France, most human infections are caused by contact with infected European brown hares (*Lepus europaeus*) [1, 9–11].

Genome sequencing and analysis have been performed on several *F*. *tularensis* strains. *Francisella s*pecies share many biological and genomic attributes [12–14]. All *Francisella* isolates have small conserved genomes of about 1.8 Mb. They have a high degree of genetic similarity and are thus monomorphic. They have average nucleotide identity (ANI) of ≥ 97.7% [15, 16] and even ≥ 99% within the subspecies *F*. *tularensis* subsp. *holarctica* [17]. However, virulence and pathogenicity are significantly influenced by slight genetic and functional differences [18–24]. Because of the limited genetic variation in *F*. *tularensis*, single nucleotide polymorphisms (SNPs) are the preferred genetic markers for molecular typing. So far, the standard of classifying sequencing data of *Francisella* [25] is a reference-dependent (full or draft genomes are used as reference) and alignment dependent (i.e. the method for calculation of a distance matrix of sequences) analysis and is termed canonical SNP typing (canSNPs). The canSNPs or other reference-dependent and alignment-dependent methods are used to unravel the relationship among of closely related populations. Additionally, a classification with canSNPs is dependent on the selected reference genomes and based on alignment and is not as mathematical independent as the methods presented. On the other hand, reference independent and alignment-independent methods exist that are not limited to a reference genome. They enable alignment of sequences without dependency on a reference genome. These methods (e.g. kSNP (reference and alignment independent), Parsnp (both reference-dependent and–independent), and average nucleotide identity (ANI) (reference and alignment independent)) and allow for resolving phylogenetic relationships, based on objective criteria.

The phylogenetic clustering reveals shared evolutionary trajectories. The most commonly used method is based on Bayesian analysis of population structure (HierBAPS), but it relies on pre-specified population structures. HierBAPS allows clustering of the phylogenetically related groups, but previous assumptions are needed [26]. HierBAPS is used as a hierarchical approach using Bayesian model-based DNA sequence clustering, where data from a cluster at a particular stage of the hierarchy are clustered in the next stage, providing a useful way of increasing statistical power to detect separate lineages residing within the data [26]. HierBAPS relies on metadata. It provides a way of recognizing similarities and differences in the distribution of similar genotypes.

To be independent of presumptions, we have devised a strategy enabling us to resolve phylogenetic cluster of bacteria also independently. The reference independent and alignment-independent method (affinity propagation clustering—APC) has successfully applied for clustering of bacterial genes and viral (Fischer, 2018) nucleotide sequences but never on whole genomes.

The aim of the study was to evaluate an analysis pipeline that utilizes a reference independent and database independent clustering method (APC), and that therefore provides a maximum level of objectivity in characterizing the phylogenetic relationships among clusters. A comprehensive panel of German *Francisella* isolates of equal quality served as an ideal data set with optimal data quality and data quantity (geographic data, PCR/qPCR and MALDI-TOF-MS)). In order to draw a comprehensive picture three different types of SNPs sets: core genome SNP, whole-genome SNP, and ANI (average nucleotide identity) were used. Together, these methods consider small, medium, and large differences. The phylogenetic clustering reveals shared evolutionary trajectories. The results were compared with the so far gold standard HierBAPS.

## Materials and methods

We assessed the phylogeny of *F*. *tularensis* subsp. *holarctica* with 152 samples from Germany and Austria collected in the years 2006–2018. The microbial phylogeny of this bacterium was assessed using assemblies from sequenced isolates. Reference genome independent methods covered the core genome, all genomic SNPs, and the ANI. First, for core genome analysis the Harvest suite was used reference independently [27]. Second kSNP3.0 was used, a k-mer based tool that can identify SNPs in hundreds of microbial genomes without the requirement for genome alignment or a reference genome [28, 29]. Third ANI analysis was done to verify the results independent of references and gene prediction. All the results were compared with laboratory methods (real-time PCR assays targeting canSNP loci) and established NGS-based methods with reference-dependent canSNPer. Then the reference-independent molecular typing tools were combined with APC, HierBAPS and SplitsTree.

### Bacterial strains

For phylogenetic analysis, bacterial strains were chosen from the collection of isolates and sequences maintained at the National Reference Laboratory of Tularemia at the Friedrich-Loeffler-Institut, Jena, Germany. *F*. *tularensis* subsp. *holarctica* strains were handled following German biosafety regulations. For this study, *F*. *tularensis* subsp. *holarctica* isolates collected in the years 2006–2018 were used and are in part publically available [1, 9, 11, 30–34]. All isolates were manually checked for completeness of metadata, e.g. year of isolation, geographical origin, and host (S1 Table, only the district is disclosed). All strains were characterized using a combination of independent methods including MALDI-TOF MS, conventional PCR and selected strains with real-time PCR assays as previously described [30, 35] (see S1 Table). Due to financial and temporal limitations, only a subset of the strains was assigned to clades using a set of real-time PCR results [10, 32]. Isolates originate from hares (*Lepus europaeus*), edible dormice *(Glis glis)*, red foxes *(Vulpes vulpes)*, and ticks *(Ixodes ricinus)*. The *F*. *tularensis* subsp. *holarctica* strains used in the present study were cultivated on cysteine heart agar (CHA, Becton Dickinson, BD Heidelberg, Germany) from animal carcasses or ticks harvested from these carcasses. The cultivation of bacteria from organ specimens was performed on cysteine heart agar at 37°C with 5% CO_2_ for 48 h. Prior to further handling isolates were inactivated at 95°C for 20 min. Sequences of reference strains NC_009749.1 (clade B.6), NC_01746 (clade B.4) and NC_019551 (clade B.12) were obtained from the NCBI database.

### DNA extraction and genome sequencing

DNA for whole-genome sequencing was prepared from a 10 ml culture in brain heart infusion broth (Sifin, Berlin, Germany). Bacterial cells were harvested after 72 h by centrifugation and the DNA was extracted and purified using QIAGEN Genomic-tip 20/G and a QIAGEN Genomic DNA buffer set kit (Qiagen, Hilden, Germany) according to the recommendation of the manufacturer. The DNA quality was examined by using a Qubit 2.0 fluorometer (Life Technologies, Darmstadt, Germany) and by agarose gel electrophoresis.

### Sequencing, assembly, annotation and genomic analysis tools

The isolates were subjected to Illumina HiSeq and/or MiSeq sequencing using the adjusted Nextera XT or HT DNA protocol for library preparation (GATC, Konstanz, Germany; BfR, Berlin, Germany or in-house). The number of reads after filtering ranged from 0.5 million to 5 million. At least 100,000 paired-end reads were generated and filtered with a Phred score averaging >38. The libraries were tested for contamination by analysis with Kraken [36] and MetaPhlAn [37]. Contaminated datasets were removed. Further processing included quality trimming and assembly (included in SPAdes 3.12.1. in Bayes Hammer mode [-—careful], [38]). Analysis of data was performed with QUAST v4.3 and Bandage 0.8.1 using standard settings [39, 40]. Filtering was performed by removing contigs with k-mer coverage less than 5x and size below 500 bases. Only assemblies without possible contamination or incomplete sequencing were allowed by excluding assemblies >4 Mb and <1 Mb of predicted total length. TempEst was used for the visualization and analysis of temporally sampled sequence data [41] as specified before [11].

### Accession numbers

New Assemblies have been stored in a BioProject that has been deposited at NCBI under the accession PRJNA560345. Data from Bioprojects PRJNA464279, PRJNA422969 and PRJNA353900 had been used.

### Phylogenetic analyses

We chose three different reference-independent methods to estimate the evolutionary distances between the investigated strains: Parsnp within the Harvest suite [27] to detect the core genome SNPs based on genome alignment, kSNP3.0 [28] to report the whole-genome SNPs in an alignment independent approach relying on kmer analysis, and the python script pyani [42] to report percentages of average nucleotide identity between the strains (S1 Fig). Parsnp and kSNP3 were used in standard settings. However, the program pyani was used with (-m ANIm) employing MUMmer (NUCmer) for alignment. From the core genome alignment produced by Parsnp, we constructed a maximum likelihood phylogeny with RaxML [43] using the GTRGAMMA model rate of heterogeneity and supported by a bootstrapping test with 100 resamples. All results of Parsnp, kSNP3.0 and pyani were compared with the laboratory typing results.

Using iTol [44] and the results from tree analysis of kSNP3.0, the distance matrices could be sorted according to the phylogenetic analysis with kSNP3.0 (S2 Fig). With core genome SNPs multiple meta-alignment files were used for SplitsTree [45]. These data were used to investigate the temporal signal of German *Francisella* in an outbreak scenario and 'clocklikeness' of molecular phylogeny using the tool TempEst results in R2 = 6,7 ∗ E-2 value, less than 0.5, suggesting weak clock-like behavior. The regression slope (rate) included negative values.

### Clustering

To group bacteria in a phylogenetically related cluster, an application of a data mining technique called affinity propagation clustering (APC) is used. This clustering technique needs no presumptions or supervision, unlike other clustering methods used for bacteria [46]. With these methods, we would like to generate new insight and a new method to cluster bacteria. APC is fast and mathematically independent. This enabled a novel detailed view on *F*. *tularensis* subsp. *holarctica* epidemiology in Germany. The APC results were compared to the results of the HierBAPS clustering [26], which needs predefined parameters. SplitsTree reveals a different angle being alignment-based but unrooted and assumption-free.

Two clustering methods were compared: APC [47] and HierBAPS [26]. APC relies on distance matrices. All three phylogenetic methods yielded distance matrices to be used in APC. Pairwise distances of 152 isolates and 3 references as calculated in the phylogenetic analysis of Parsnp, kSNP3.0, and pyani were merged into a distance matrix each and imported to the statistics software R [48]. For further analysis, the package “apcluster” was used essentially as described. By default, the APC algorithm determines one sequence among the set of input elements for each potential cluster, which is most representative of this cluster. In APC terminology, these sequences are termed “cluster exemplars”. Since this method was initially developed for the analysis of similarity matrices, the distance matrix from the sequence alignment had to be converted by inverting the values. Also, all values embedded in the matrix were squared to improve the robustness and discriminatory power of the analysis. Subsequently, the APC algorithm computes the minimum (pmin) and maximum (pmax) of the input preference (p), which is defined as the tendency of each sequence to become a “cluster exemplar” [47]. To define the optimal input preference (p), the number of cluster for the complete preference range (pmin-pmax) was calculated stepwise. The optimal input parameter for intraspecific analyses, i.e. the optimal number of cluster, was defined as the largest range of input parameters for which a constant number of cluster is calculated. This range is termed “plateau” throughout the manuscript. Methodologically, the beginning of the lower bound of the “two cluster plateau” cannot be defined and therefore the length of this plateau was not considered further. According to the now defined optimum input parameter, APC calculated the respective number of cluster and allocated any input sequence to only one of these. Results of APC were summarized on the country level and exported as CSV file into the GIS software QGIS (Version 2.18 "Las Palmas"; QGIS Development Team (2019)). QGIS Geographic Information System. Open Source Geospatial Foundation Project. http://qgis.osgeo.org). Each sequence dataset was completed with the assigned clade using PCR/qPCR results.

As comparison HierBAPS in the standard-setting was used [26]. As an unrooted phylogenetic network SplitsTree [45] with standard-setting using the aligned SNPs from Parsnp. The statistics program silhouette analysis was used to evaluate the separation distance between the resulting cluster.

## Results

In total, 152 *F*. *tularensis* subsp. *holarctica* isolates were sequenced and assembled to assess the phylogenetic relationships between them. Three reference sequences, showing the three major clades of *F*. *tularensis* subsp. *holarctica* were added from the NCBI database (NC_009749, NC_017463, NC_019551). After quality and contamination filtering (excluding data with a PHRED score >38 and contamination of more than 20% reads from other species). The quality assessment showed that individual sequence lengths of the assemblies were 1.8 million nucleotides derived from 79 to 145 contigs. Altogether the sequences had an average G+C content of 32.2%.

### Phylogenetic analysis

**Fig 1:**
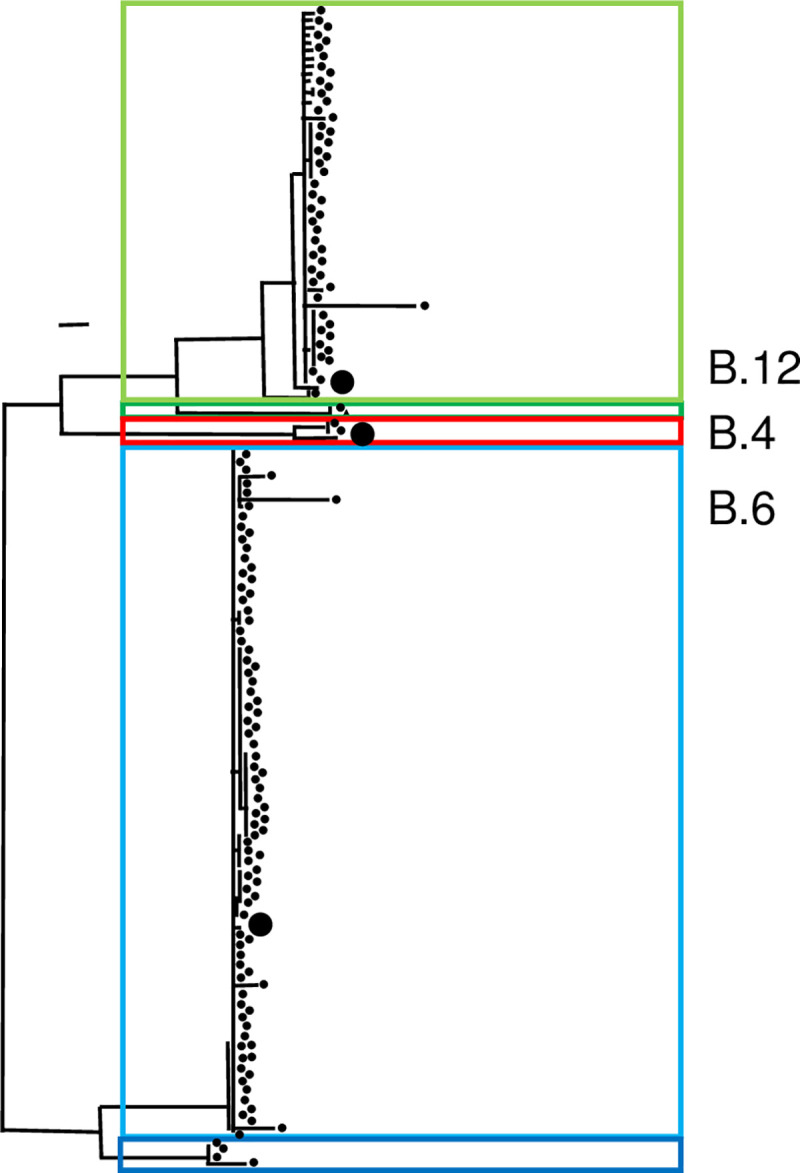
Phylogeny-based identified core genome SNP and phylogenetic analysis, performed with the Harvest suite. Red lines indicate assignment to clade B.4, green to clade B.12, dark green subclade B.71, blue to clade B.6 and dark blue subclade B.7.

First, a core genome analysis was conducted with the Parsnp [26] (Fig 1). The mandatory ≥ 97% ANI was verified (S1 Fig). Using the phylogenetic analysis three cluster could be visually distinguished. These cluster are in concordance with clades analyzed in the laboratory by PCR as B.4, B.6 and B.12 (Clade, PCR verified, S1 Table). Additionally, the subclade B.7 and B.71 within the clades B.6 and B.12 (with the subclade definition used by the canSNPer and the real-time PCR) could be visually distinguished and verified.

**Fig 2:**
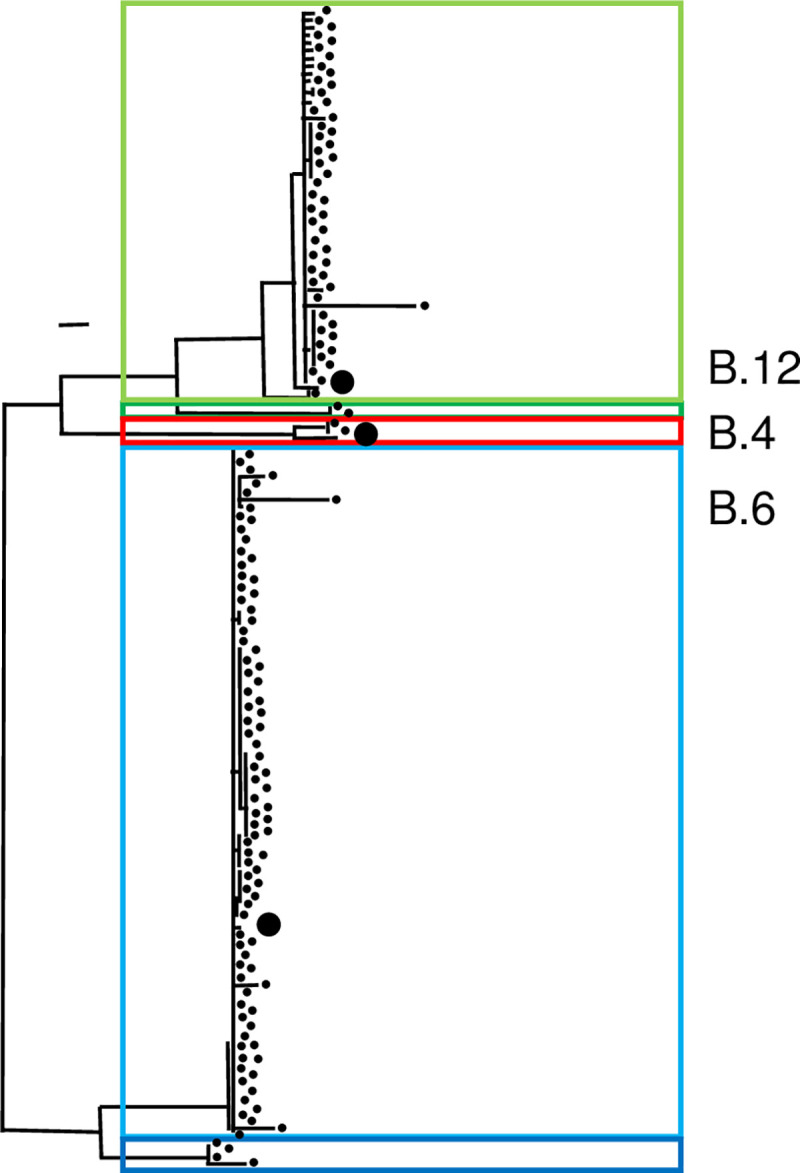
Phylogeny-based on kSNP3.0: Identified SNPs and analyzed phylogeny without genome alignment or the requirement of a reference genome. Red lines indicate assignment to clade B.4, green to clade B.12, dark green subclade B.71, blue to clade B.6 and dark blue subclade B.7.

Second, within the maximum likelihood tree of kSNP3.0, the three cluster described above were present. In addition, two subclades B.7 and B.71 could be distinguished by trend (Fig 2). The kSNP3.0 analysis was followed by RaxML. For the parsimony analysis, 3,985 SNPs were used. In these analyses, 97.1% (299 out of 308) of all nodes showed bootstrap values higher than 70%. One visible subcluster within the cluster B.6 correlated to the canSNPer classification of B.7. The other potential subcluster within the subgroup B.12 correlates to the canSNPer classification of B.71. Not in all cases, canSNPer analysis correlated with the resulting three phylogenetic analyses described. In particular, the subcluster groups containing isolates B.45 and B.61 appear to be much more divergent. The subclades within the clade B.12 do not correlate continuously. KSNP3.0 reports a consensus of the equally most parsimonious trees. In total, 3,985 SNPs were reported for all genomes, compared to the 116 SNPs reported in the canSNPer (version Wittwer 2018).

As a third method, the determination of the ANI was used. The ANI of all isolates was extremely high identical within the isolates with more than 99.9% identity. The three clades mentioned above could be distinguished and to a lesser extent B.7 and B.71.

**Fig 3:**
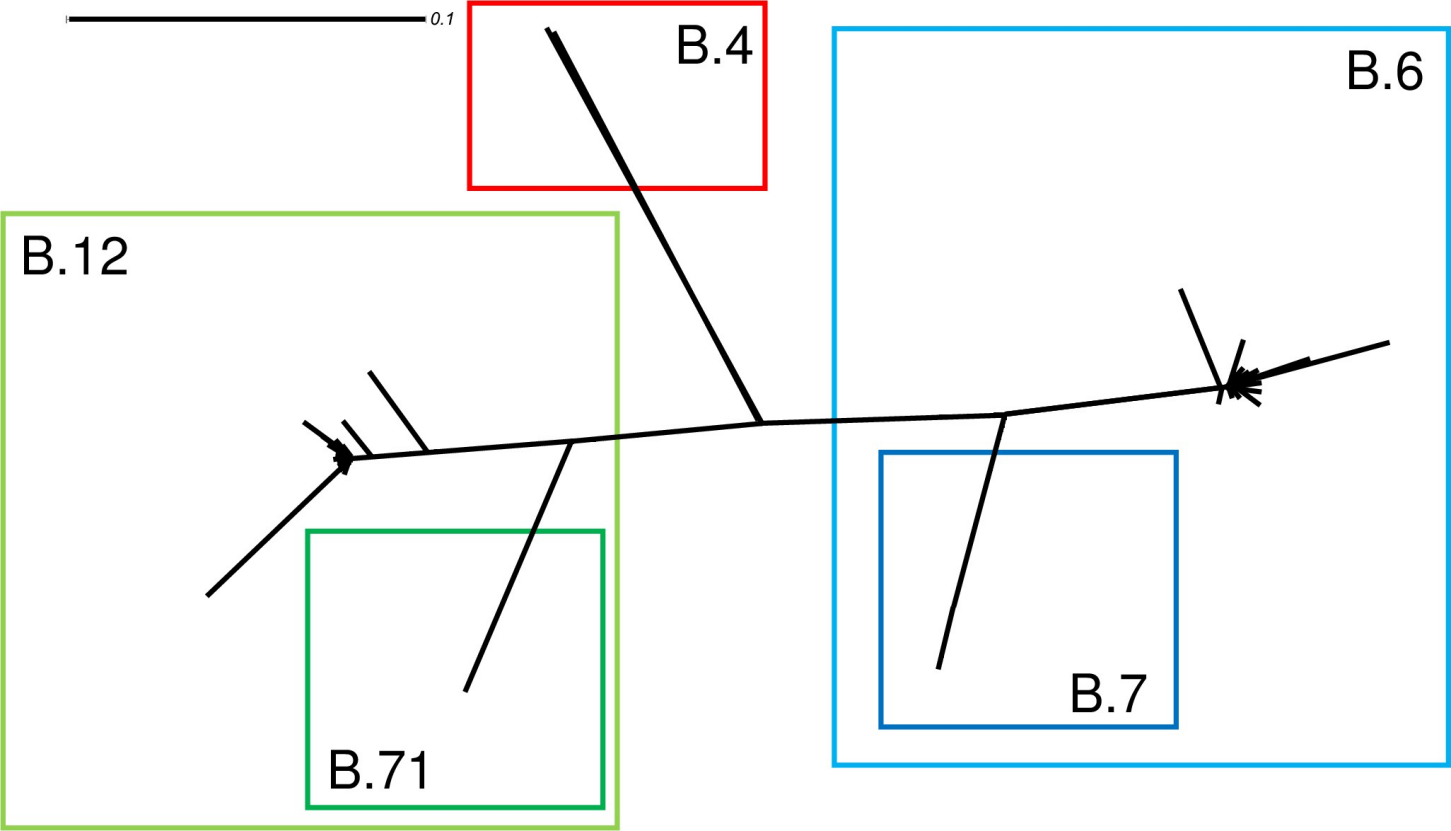
An unrooted phylogenetic network SplitsTree. Red lines indicate assignment to clade B.4, green to clade B.12, dark green subclade B.71, blue to clade B.6 and dark blue subclade B.7.

SplitsTree, an alignment-dependent but assumption-free method was used to generate unrooted phylogenetic networks from molecular sequence data. Given an alignment of core-genomic SNPs extracted from the Harvest suite, the program computed a network (Fig 3) and confirmed the three cluster (B.4, B.6, B.12) and to a lesser extent B.7 and B.71.

### Clustering analysis

**Fig 4:**
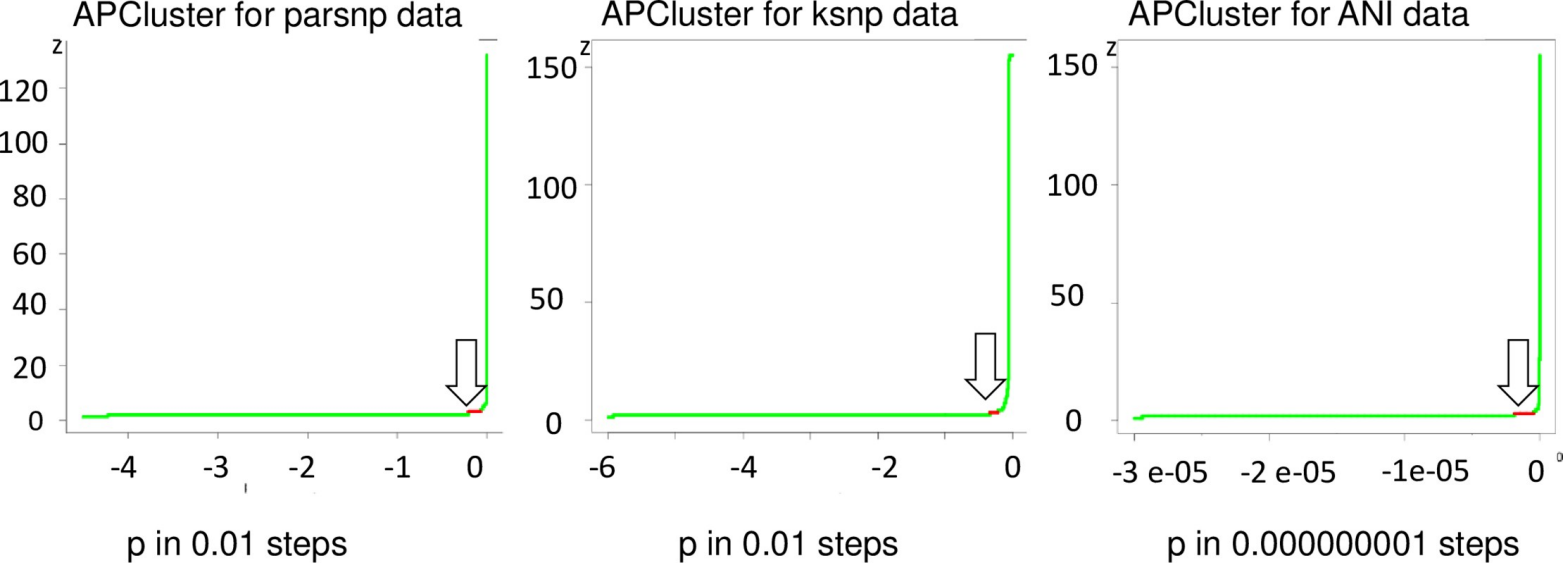
Graphical display of affinity propagation clustering over the range of all included samples and references. Optimal input preference for intraspecific analyses, i.e. the optimal number of cluster, was defined as the largest plateau (three AP cluster, see arrows). The y-axis represents the number of cluster while the x-axis represents the input parameter. A: based on core genome SNPs, B: based on whole-genome SNPs and C: based on ANI. Optimal input preference for intraspecific analyses, i.e. the optimal number of cluster, was defined as the largest plateau (three AP cluster) as methodologically, the beginning of the lower bound of the two cluster plateau cannot be defined. (Preference range ANI: between -3e-05 and 0; preference range kSNP: between -6 and 0; preference range Parsnp: between -4.5 and 0).

A dendrogram does not allow to answer the question if we should distinguish between three (B.4, B.12, And B.) or five clades (additionally B.71 and B.7). Dendrograms remain subjective. The clustering analysis provided an objective way to calculate of how many cluster are in a sample set. As a central result, we could define a three cluster demarcation within the *F*. *tularensis* subsp. *holarctica* isolates in Germany. APC assigned three genetic cluster in all distance matrices generated by the three methods (Parsnp, kSNP3.0, pyani) (Fig 4) and was comparable to real-time PCR assays in the laboratory. APC has a high computational efficiency [49], which substantially reduces the turnaround time. The main advantage is that it overcomes the described subjective criteria for cluster allocations with the help of mathematical algorithms. The results are non-hierarchically ordered cluster [49, 50]. By application of APC to pairwise genetic distances from an alignment of all 152 + 3 sequences, the most stable distribution after iteration over all possible input parameters was determined as three cluster (Fig 4). Statistic support was calculated with the r package silhouette. The analysis calculated the separation distance between the resulting cluster. For all three matrices the average silhouette width with strongest support (0,71–1) (Parsnp: kmeans 0.96/pam 0.92, KSNP3.0: kmeans 0.83/pam 0.83, ANI: kmeans 0.75/pam 0.76). Three plateaus had the highest support in the proportional distribution (S4 Table). These are in accordance with the cluster B.4, B.6, and B.12 as tested before (S1 Table and S3 Table).

As another clustering method, HierBAPS is compared to APC. HierBAPS is not mathematical independent as affinity propagation. HierBAPS is used to detect the underlying population substructure dependent on SNP alignment, which is done by Parsnp. This approach involves the application of iterative clustering [47, 51]. The software HierBAPS identified four major cluster at the first level of clustering and nine cluster at the second clustering level (S2 Table). The analysis of the distinguishing core genome SNPs from Parsnp alignment showed in each cluster a dramatic reduction of the SNPs (from 1,517 to 371, 358 and 65) by supporting the formerly obtained results. The subclades B.7 and B.71 showed a less dramatic reduction compared to the clades with 37 and 9 SNPs to their clades, respectively (S2 Table). The grouping of subpopulations by HierBAPS correlates with the other results; however, one of the subcluster identified by HierBAPS at the first partitioning level included members of B.4 and the subcluster B.71 in affinity propagation, whereas they belonged to clade B.12 based on laboratory methods and APC (S1 Table).

The maximal geographic distance of isolates of one indistinguishable branch is 275–350 km (09T0105, 09T0115, 09T0167, 11T0126, 11T0309, 17T1184 and 15T0759, 11T0023, 14T0103, and 14T0102). The maximal geographic distance of isolates of the subcluster residing within B.12, B.71, is ca. 400 km.

**Fig 5:**
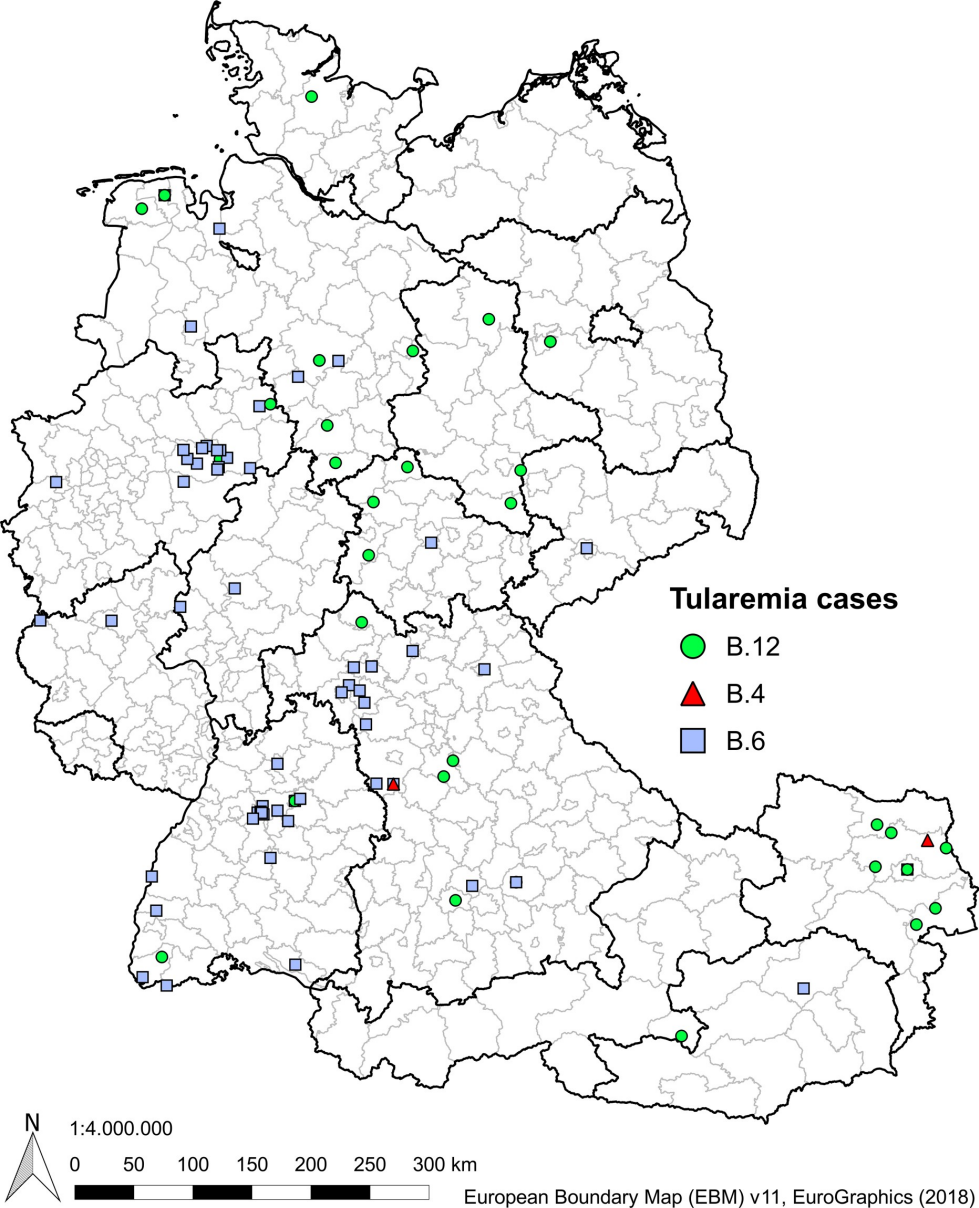
Geographical representation of the genetic population structure of *Francisella tularensis* subsp. *holarctica* isolates in Germany and Austria. Icons are actual cases of infection with a geotag from which the isolation occurred. Red dots indicate assignment to clade B.4, green to clade B.12, dark green subclade B.71, blue to clade B.6, and dark blue subclade B.7. (Version 2.18 "Las Palmas"; QGIS Development Team (2019). QGIS Geographic Information System. Open Source Geospatial Foundation Project. http://qgis.osgeo.org).

The geographical representation of all isolates is displayed with the APC designation (Fig 5). The data provide evidence that Germany is indeed the collision zone were the clade B.12, also known as East European clade overlaps with the clade B.6, also known as Iberian clade [1, 2].

## Discussion

Whole-genome sequencing can be used for bacterial strain typing and epidemiologic analysis [52, 53]. A whole array of bioinformatics tools are available for processing NGS data are increasingly open-source and of high quality [53–55]. There are two main analytical bioinformatics approaches to the exploitation of whole-genome sequencing data: reads can be aligned or subjected to *de novo* assembly [56]. The choice of strategy depends on the read length obtained, the availability of good reference sequences and the intended biological application. Alignment-based sequence comparison has several setbacks: alignment-producing programs assume collinearity, which is very often violated in the real world. The accuracy of sequence alignments drops off rapidly in cases where the sequence identity falls below a certain critical point, the generation of alignments is memory and time-consuming, an NP-hard problem and depends on multiple *a priori* assumptions about the evolution of the sequences that are being compared. For these reasons, we tested alignment-free assembly-based methods. Since 2012 *de novo* assembly strategies have progressed greatly [57]. The advantage of using assemblies is that there is no bias towards a reference genome. In addition, no template has to be adapted, and though it needs more reads and it is generally more fragmented, it works better for large-scale and medium-scale differences. Mapping can be better for small-scale differences such as SNPs and small variations, however, new sequences that are completely different can be lost.

We tested and compared methods that are either dependent or independent of a reference of an assembly or an alignment. With a growing number of genome sequences the multiple alignments of homologous sequences followed by inference of a tree scale poorly. Therefore, independent ‘alignment-free’ methods should be preferably used (71). We aimed for a universally applicable workflow to analyze phylogenetic cluster of bacteria using *Francisella tularensis* subsp. *holarctica* as a model organism.

When using genomic variants for phylogenetic analysis, comparative genomics, or outbreak investigations, it is critical to properly evaluate the variant calling method and to re-evaluate them regularly. Core genome SNPs (Parsnp), whole-genome SNPs (kSNP3.0) and ANI (pyani) were compared to each other.

Parsnp combines the advantages of both, whole-genome alignment, read mapping, and can be scaled to thousands of closely related genomes. To achieve this scalability, Parsnp is based on a suffix graph data structure for the rapid identification of maximal unique matches (MUMs), which serve as a common foundation for many pairwise and multiple genome alignment tools. The core genome of the genome sequences is used to create multiple meta-alignment files (used in Parsnp analysis (26) and SplitsTree (46)). In the unrooted network analysis of SplitsTree, the three clades of *Francisella* isolates were independent of the rooted phylogeny distinguishable, reinforcing the made assumption.

kSNP3.0 is a program for SNP identification and phylogenetic analysis without genome alignment or the requirement for reference genomes. kSNP3.0 is based on k-mer recognition. It provides the highest resolution compared to canSNPer, Parsnp, and pyani. The kSNP3.0 analysis allowed deciphering even quite subtle signals of systematic differences in molecular variation (Fig 2) comparable to the Parsnp analysis. A direct comparison to the canSNPer analysis that allows discreet, though more subjective subclustering could be generated (S1 Table, S3 Fig). The bootstrap values of more than 70% are recognized as a threshold for reliability [58], demonstrating that bootstrap support of nodes does not result in the delineation of meaningful cluster, if large numbers of full genome sequences are analyzed [59]. The kSNP3.0 allowing an analysis of at least 34–fold higher resolution than canSNPer. The higher resolution assigned only 3 cluster compared to more than 80 in the canSNPer. Three cluster could be validated with three independent methods and physiological relationships (for example erythromycin resistance [60]). It would be interesting to see if with an independent method the canSNPer designs really show relevant cluster and physiological relationships. It might be overdesigning the cluster. Nevertheless, all used data correlated mainly with the phenotype, canSNPer, and qPCR results (S3 Fig).

The ANI is a measure of nucleotide-level genomic similarity especially suitable for major differences. Although *Francisella* is highly monomorphic, the clustering approach could be verified in a reference-independent and alignment-free independent method.

The aim of this study was to resolve if it is possible to determine phylogenetic relationships and clustering of bacteria to optimize outbreak analysis for *Francisella tularensis* isolates in Germany. Classification, clustering, and designation is a key step in phylogenetic and outbreak analysis required for understanding and adequate management of organisms.

The familiar evolutionary model assumes a tree, a model that has greatly facilitated the discussion and testing of hypotheses. However, it is well known that such models (46) poorly describe more complex evolutionary scenarios. Therefore, we used SplitsTree in a combination with the core genome alignment of Parsnp to visualize cluster within an unrooted model. We added thus an independent approach to the phylogenetic analysis and verified the three detected cluster. In addition, the subclades B.7 and B.71 were distinguishable (S4 Fig).

After phylogenetic analysis, a clustering is needed to allow cluster definition root-independent and objectively. Two clustering methods were compared: affinity propagation and HierBAPS. With APC it is not relevant to know the evolutionary dynamics of *F*. *tularensis* subsp. *holarctica* and it needs no further preconceptions as in HierBAPS. Affinity propagation provided meaningful clustering. It can be performed on whole-genome sequences [50, 61].

We found that APC is particularly suitable for applications in clustering bacteria [47]. APC is a tool that was developed for clustering similarity measures between all pairs of input samples based on the concept of "message passing" between data points. Besides its computational efficiency, which substantially reduces the turnaround time, the main advantage is the overcoming of the described subjective criteria for cluster allocations with the help of mathematical algorithms. The results are non-hierarchically ordered cluster, which can have unequal cluster sizes [49, 50]. Thus subjective cluster allocation can be made. By application of APC to pairwise genetic distances from an alignment of *F*. *tularensis* subsp. *holarctica* genomes, the most stable distribution after iteration over all possible input parameters were determined and three cluster equal to known clades were defined. This approach has already been successfully applied for various tasks in bioinformatics, e.g. for microarray and gene expression data [62–65] but not to our knowledge on whole-genome analysis. An extended panel of newly obtained full genomes sequences of *F*. *tularensis* subsp. *holarctica* was used to demonstrate the application of APC for bacteria and thus clade definition as well as for comparison with previous studies. Similar problems have been described for viruses and bacteria of other genera and alternative solutions have been developed [58, 66, 67]. APC seems particularly promising and could help to solve species delineations in asexual lineages where obligate gene exchange cannot be used as a delineation criterion [61]. The high speed of the APC algorithm gives the possibility to analyze very large data sets. We, therefore, believe that this algorithm is very useful for classifying cluster in other bacteria. The results were confirmed with the statistical analysis program silhouette (r-package cluster 2.1.0, [68]) to verify the cluster number and showing that the clustering had strong support.

We confirmed the reliability of the subclustering also with independent mathematical methods such as SplitsTree and HierBAPS. The clustering of the affinity propagation was verified with an independent method, HierBAPS [26] (S3 Table).

As comparison HierBAPS in the standard-setting was used as a hierarchical approach using Bayesian model-based DNA sequence clustering. Data from a cluster at a particular stage of the hierarchy are reclustered in the next stage, providing a useful way of increasing statistical power to detect separate lineages residing within the data [26]. A parallel approach was an unrooted phylogenetic network. The results of BAPS also n on the phylogenetic results when taking into account the first and second levels of BAPS clustering. The discovery of nested genetic population structures has been shown before [51]. Four clades were identified into two subcluster. The software HierBAPS split cluster B.4 and the subcluster equivalent to subcluster B.71, traditionally belonging to B.12, in the second level of clustering. One can assume that one of these clades showed more divergence between the strain, which could be divided into two subcluster in the second partitioning of the clades. This is a known issue for the BAPS algorithm, where a conservative clustering is applied. However; the population sample could not be uniformly divergent [26]. When we consider the second level of clustering, five clades and four outliers could be delineated, supporting the results of APC of three clades B.4, B.6, and B.12, and the subclades B.7 and B.71. The clustering algorithm is also known to benefit from large sample numbers and improve with more samples, so when more data are provided, the results might concur with the laboratory data. The cluster identified by APC was compared to HierBAPS. Both methods describe qualitatively distinct clades. HierBAPS’ clusters are not preferable to APCluster’s; because the algorithm needs preconceptions. The cluster are not in concordance with the canSNPer results and the computing time is longer. APC has the advantage that a mathematical independent objectively approach, is in consensus with laboratory data and has fast computing times.

For outbreak analysis, it is imperative to analyze the host, temporal and phylogeographic association within the underlying phylogenetic analysis. There is a strong indication of the correct assignment and support for the methods used (core genome SNP, whole-genome SNP, and ANI). For the host association not distinguishable, unfortunately, no confirmation of the route of infection tick-hare or hare-tick could be confirmed. The hosts of the subcluster B.7 were two edible dormice, a hare, and a fox showing an unusual host preference, bearing in mind that hares that were host to over 90% of the isolates in this data set. The low diversity of the dataset could lead to a biased interpretation and might be remedied in time. For the temporal and phylogeographic association, only nidal and random temporal and spatial distribution patterns can be described. To assess the temporal evolution the rates of molecular evolution are calculated by the product of the number of mutations that arise per replication event, the frequency of replication events per unit time and the probability of mutational fixation [69]. The regression slope (rate) found included negative values, suggesting that these rates are either too low or not enough with the used data set to allow reliable rate estimation. Similar data are obtained from *Mycobacterium leprae*. This suggests that the high specialization and success of *F*. *tularensis* subsp. *holarctica* leads to an evolutionary dead-end such as in *Clostridium chauvoei* [70]. An analysis with BEAST, as done before with only 67 samples [2] is not recommended, because the molecular clock models employed are statistically conditioned on having an evolutionary rate greater than zero [41]. That might be due to fewer samples or to a different assembler strategy, that had been shown to an over-and underestimated gene content and genome sizes up to 36% [11].

In time the sample number of isolates will be more comprehensive. When used with more isolates from different countries it might be even possible to establish a higher spatial and temporal resolution and thus to generate a highly standardized nomenclature for subpopulations. The geographic resolution is allowed within limits of a 400 km radius. However, for clonal organisms as *Francisella*, it can be difficult to decide where to set demarcations between groups [15, 54, 56, 60, 61, 71–74]. The geographic distance of indistinguishable isolates can be 275–400 km demonstrates the wide distribution of *F*. *tularensis* subsp. *holarctica* within Germany. The data provide evidence that Germany is indeed the collision zone were the clade B.12, also known as the East European clade overlaps with the clade B.6, also known as Iberian clade. In previous phylogenetic studies, cluster allocation of *Francisella* isolates was either based on qPCR, PCR, canSNPer, and region of origin. In the epidemiological collision zone, where the Clade B.6 and B.12 meet a boundary is perceivable. At this boundary changes of clades can occur in the spatial distribution (for example migration) and genetic changes (for example transfer of genetic elements). This should be carefully monitored. However, the allocation of *F*. *tularensis* subsp. *holarctica* into clades might biased and subjective because the thresholds of statistical support vary depending on the respective reference genomes and databases. Though one of the major subcluster B.7 and B.71 correlate well with traditional methods, further split subclades reflect the results from canSNPer and qPCR could not be consistently verified. Combining cluster identification, we could provide a simplified evolutionary scheme for *F*. *tularensis* subsp. *holarctica*. By applying affinity propagation on whole genomes of other bacteria, other clades and subclades in these pathogens will be accessible cost-efficient and free software. This should allow the rapid risk assessment in the setting of epidemics and outbreaks.

## Supporting information

S1 Fig Matrix-based on the identity of average nucleotide identity (ANI). Background coloring on the percentage of identity from green (1) to red (<0,9998). Red lines indicate assignment to clade B.4, green to clade B.12, dark green subclade B.71, blue to clade B.6 and dark blue subclade B.7
(TIF)

S2 Fig Phylogenetic tree based on average nucleotide identity (ANI). (TIF)

S3 Fig kSNP3.0 neighbor-joining tree with canSNPer results. (TIF)

S4 Fig An unrooted phylogenetic network SplitsTree. Red lines indicate assignment to clade B.4, green to clade B.12, dark green subclade B.71, blue to clade B.6 and dark blue subclade B.7, canSNPer data included.
(TIF)

S1 Table *Francisella tularensis* subsp. *holarctica* isolates from Germany with qPCR and canSNPer results, year of collection, and district of isolation. (x; no result obtained). Isolates hosted by hare and ticks belonging together are defined as follows: 10T0153, 10T0156; 11T0041, 11T0126; 11T0305, 11T0309; 11T0313, 11T0315, 11T0316, 11T0319; 12T0017, 12T0020, 12T0021, 12T0022; 13T0041, 13T0053, 13T0054.
(DOCX)

S2 Table Analysis of SNP numbers that distinguish the cluster. (DOCX)

S3 Table Statistical analysis of clustering independent of SNP Typing with hierBAPS. (DOCX)

S4 Table The possible plateaus and their proportional distribution in percent. (DOCX)

## References

[1] Müller W, Hotzel H, Otto P, Karger A, Bettin B, Bocklisch H, et al German Francisella tularensis isolates from European brown hares (Lepus europaeus) reveal genetic and phenotypic diversity. BMC Microbiol. 2013;13:61 Epub 2013/03/23. 10.1186/1471-2180-13-61 23517149 PMC3663675

[2] Dwibedi C, Birdsell D, Larkeryd A, Myrtennas K, Ohrman C, Nilsson E, et al Long-range dispersal moved Francisella tularensis into Western Europe from the East. Microbial genomics. 2016;2(12):e000100 Epub 2017/03/30. 10.1099/mgen.0.000100 28348839 PMC5359409

[3] Ellis J, Oyston PC, Green M, Titball RW. Tularemia. Clin Microbiol Rev. 2002;15(4):631–46. Epub 2002/10/05. 10.1128/cmr.15.4.631–646.2002 12364373 PMC126859

[4] Hirschmann JV. From Squirrels to Biological Weapons: The Early History of Tularemia. The American journal of the medical sciences. 2018;356(4):319–28. Epub 2018/08/28. 10.1016/j.amjms.2018.06.006 . 30146078

[5] Bröms JE, Sjöstedt A, Lavander M. The Role of the Francisella Tularensis Pathogenicity Island in Type VI Secretion, Intracellular Survival, and Modulation of Host Cell Signaling. Front Microbiol. 2010;1:136 Epub 2010/01/01. 10.3389/fmicb.2010.00136 21687753 PMC3109350

[6] Rotz LD, Khan AS, Lillibridge SR, Ostroff SM, Hughes JM. Public health assessment of potential biological terrorism agents. Emerg Infect Dis. 2002;8(2):225–30. Epub 2002/03/19. 10.3201/eid0802.010164 11897082 PMC2732458

[7] Kingry LC, Petersen JM. Comparative review of Francisella tularensis and Francisella novicida. Front Cell Infect Microbiol. 2014;4:35 Epub 2014/03/25. 10.3389/fcimb.2014.00035 24660164 PMC3952080

[8] Tarnvik A, Berglund L. Tularaemia. Eur Respir J. 2003;21(2):361–73. Epub 2003/03/01. 10.1183/09031936.03.00088903 . 12608453

[9] Otto P, Kohlmann R, Muller W, Julich S, Geis G, Gatermann SG, et al Hare-to-human transmission of Francisella tularensis subsp. holarctica, Germany. Emerg Infect Dis. 2015;21(1):153–5. Epub 2014/12/23. 10.3201/eid2101.131837 25531286 PMC4285259

[10] Robert-Koch-Institut. Infektionsepidemiologisches Jahrbuch meldepflichtiger Krankheiten für 2015. Infektionsepidemiologisches Jahrbuch. 2015.

[11] Busch A, Thomas P, Zuchantke E, Brendebach H, Neubert K, Gruetzke J, et al Revisiting Francisella tularensis subsp. holarctica, Causative Agent of Tularemia in Germany With Bioinformatics: New Insights in Genome Structure, DNA Methylation and Comparative Phylogenetic Analysis. Frontiers in microbiology. 2018;9:344 Epub 2018/03/30. 10.3389/fmicb.2018.00344 29593661 PMC5859110

[12] Jones BD, Faron M, Rasmussen JA, Fletcher JR. Uncovering the components of the Francisella tularensis virulence stealth strategy. Frontiers in cellular and infection microbiology. 2014;4:32 Epub 2014/03/19. 10.3389/fcimb.2014.00032 24639953 PMC3945745

[13] Ulland TK, Janowski AM, Buchan BW, Faron M, Cassel SL, Jones BD, et al Francisella tularensis live vaccine strain folate metabolism and pseudouridine synthase gene mutants modulate macrophage caspase-1 activation. Infection and immunity. 2013;81(1):201–8. Epub 2012/11/02. 10.1128/iai.00991-12 23115038 PMC3536133

[14] Koene M, Rijks J, Maas M, Ruuls R, Engelsma M, van Tulden P, et al Phylogeographic Distribution of Human and Hare Francisella Tularensis Subsp. Holarctica Strains in the Netherlands and Its Pathology in European Brown Hares (Lepus Europaeus). Frontiers in cellular and infection microbiology. 2019;9(11). 10.3389/fcimb.2019.00011 PMC6378916 30805312

[15] Larsson P, Elfsmark D, Svensson K, Wikstrom P, Forsman M, Brettin T, et al Molecular evolutionary consequences of niche restriction in Francisella tularensis, a facultative intracellular pathogen. PLoS Pathog. 2009;5(6):e1000472 Epub 2009/06/13. 10.1371/journal.ppat.1000472 19521508 PMC2688086

[16] Champion MD. Host-pathogen o-methyltransferase similarity and its specific presence in highly virulent strains of Francisella tularensis suggests molecular mimicry. PloS one. 2011;6(5):e20295 Epub 2011/06/04. 10.1371/journal.pone.0020295 21637805 PMC3102702

[17] Vogler AJ, Birdsell D, Price LB, Bowers JR, Beckstrom-Sternberg SM, Auerbach RK, et al Phylogeography of Francisella tularensis: global expansion of a highly fit clone. J Bacteriol. 2009;191(8):2474–84. Epub 2009/03/03. 10.1128/jb.01786-08 19251856 PMC2668398

[18] Johansson A, Goransson I, Larsson P, Sjostedt A. Extensive allelic variation among Francisella tularensis strains in a short-sequence tandem repeat region. J Clin Microbiol. 2001;39(9):3140–6. Epub 2001/08/30. 11526142 10.1128/JCM.39.9.3140-3146.2001 PMC88310

[19] Beckstrom-Sternberg SM, Auerbach RK, Godbole S, Pearson JV, Beckstrom-Sternberg JS, Deng Z, et al Complete genomic characterization of a pathogenic A.II strain of Francisella tularensis subspecies tularensis. PLoS One. 2007;2(9):e947 Epub 2007/09/27. 10.1371/journal.pone.0000947 17895988 PMC1978527

[20] Chaudhuri RR, Ren CP, Desmond L, Vincent GA, Silman NJ, Brehm JK, et al Genome sequencing shows that European isolates of Francisella tularensis subspecies tularensis are almost identical to US laboratory strain Schu S4. PLoS One. 2007;2(4):e352 Epub 2007/04/05. 10.1371/journal.pone.0000352 17406676 PMC1832225

[21] Barabote RD, Xie G, Brettin TS, Hinrichs SH, Fey PD, Jay JJ, et al Complete genome sequence of Francisella tularensis subspecies holarctica FTNF002-00. PLoS One. 2009;4(9):e7041 Epub 2009/09/17. 10.1371/journal.pone.0007041 19756146 PMC2737636

[22] Johansson A, Petersen JM. Genotyping of Francisella tularensis, the causative agent of tularemia. J AOAC Int. 2010;93(6):1930–43. Epub 2011/02/15. . 21313823

[23] Larson MA, Nalbantoglu U, Sayood K, Zentz EB, Bartling AM, Francesconi SC, et al Francisella tularensis Subtype A.II Genomic Plasticity in Comparison with Subtype A.I. PLoS One. 2014;10(4):e0124906 Epub 2015/04/29. 10.1371/journal.pone.0124906 25918839 PMC4412822

[24] Chen F, Rydzewski K, Kutzner E, Hauslein I, Schunder E, Wang X, et al Differential Substrate Usage and Metabolic Fluxes in Francisella tularensis Subspecies holarctica and Francisella novicida. Front Cell Infect Microbiol. 2017;7:275 Epub 2017/07/07. 10.3389/fcimb.2017.00275 28680859 PMC5478678

[25] Svensson K, Back E, Eliasson H, Berglund L, Granberg M, Karlsson L, et al Landscape epidemiology of tularemia outbreaks in Sweden. Emerg Infect Dis. 2009;15(12):1937–47. Epub 2009/12/08. 10.3201/eid1512.090487 19961673 PMC3044527

[26] Cheng L, Connor TR, Siren J, Aanensen DM, Corander J. Hierarchical and spatially explicit clustering of DNA sequences with BAPS software. Molecular biology and evolution. 2013;30(5):1224–8. Epub 2013/02/15. 10.1093/molbev/mst028 23408797 PMC3670731

[27] Treangen TJ, Ondov BD, Koren S, Phillippy AM. The Harvest suite for rapid core-genome alignment and visualization of thousands of intraspecific microbial genomes. Genome Biol. 2014;15(11):524 Epub 2014/11/21. 10.1186/preaccept-2573980311437212 25410596 PMC4262987

[28] Gardner SN, Slezak T, Hall BG. kSNP3.0: SNP detection and phylogenetic analysis of genomes without genome alignment or reference genome. Bioinformatics. 2015;31(17):2877–8. Epub 2015/04/29. 10.1093/bioinformatics/btv271 . 25913206

[29] Hall BG. SNP-associations and phenotype predictions from hundreds of microbial genomes without genome alignments. PloS one. 2014;9(2):e90490 Epub 2014/03/04. 10.1371/journal.pone.0090490 24587377 PMC3938750

[30] Tomaso H, Scholz HC, Neubauer H, Al Dahouk S, Seibold E, Landt O, et al Real-time PCR using hybridization probes for the rapid and specific identification of Francisella tularensis subspecies tularensis. Mol Cell Probes. 2007;21(1):12–6. Epub 2006/08/09. 10.1016/j.mcp.2006.06.001 . 16893624

[31] Busch A, Thomas P, Myrtennas K, Forsman M, Braune S, Runge M, et al High-Quality Draft Genome Sequence of Francisella tularensis subsp. holarctica Strain 08T0073 Isolated from a Wild European Hare. Genome Announc. 2017;5(12). Epub 2017/03/25. 10.1128/genomeA.01577-16 28336603 PMC5364228

[32] Tomaso H, Otto P, Peters M, Suss J, Karger A, Schamoni H, et al Francisella tularensis and other bacteria in hares and ticks in North Rhine-Westphalia (Germany). Ticks Tick Borne Dis. 2017 Epub 2017/12/15. 10.1016/j.ttbdis.2017.11.007 . 29239792

[33] Tomaso H, Otto P, Peters M, Suss J, Karger A, Schamoni H, et al Francisella tularensis and other bacteria in hares and ticks in North Rhine-Westphalia (Germany). Ticks and tick-borne diseases. 2018;9(2):325–9. Epub 2017/12/15. 10.1016/j.ttbdis.2017.11.007 . 29239792

[34] Kammeyer P, Hartmann P., Busch A., Tomaso H., Braune S., Runge M., Kleinschmidt S. Glis glis–two cases of acute tularemia. Berl Münch Tierärztl Wochenschr. 2019 10.2376/0005-9366-18080

[35] Larkeryd A, Myrtennas K, Karlsson E, Dwibedi CK, Forsman M, Larsson P, et al CanSNPer: a hierarchical genotype classifier of clonal pathogens. Bioinformatics. 2014;30(12):1762–4. Epub 2014/02/28. 10.1093/bioinformatics/btu113 . 24574113

[36] Wood DE, Salzberg SL. Kraken: ultrafast metagenomic sequence classification using exact alignments. Genome Biol. 2014;15(3):R46 Epub 2014/03/04. 10.1186/gb-2014-15-3-r46 24580807 PMC4053813

[37] Segata N, Waldron L, Ballarini A, Narasimhan V, Jousson O, Huttenhower C. Metagenomic microbial community profiling using unique clade-specific marker genes. Nat Methods. 2012;9(8):811–4. Epub 2012/06/13. 10.1038/nmeth.2066 22688413 PMC3443552

[38] Bankevich A, Nurk S, Antipov D, Gurevich AA, Dvorkin M, Kulikov AS, et al SPAdes: a new genome assembly algorithm and its applications to single-cell sequencing. J Comput Biol. 2012;19(5):455–77. Epub 2012/04/18. 10.1089/cmb.2012.0021 22506599 PMC3342519

[39] Gurevich A, Saveliev V, Vyahhi N, Tesler G. QUAST: quality assessment tool for genome assemblies. Bioinformatics (Oxford, England). 2013;29 10.1093/bioinformatics/btt086 PMC3624806 23422339

[40] Wick RR, Schultz MB, Zobel J, Holt KE. Bandage: interactive visualization of *de novo* genome assemblies. Bioinformatics. 2015;31(20):3350–2. Epub 2015/06/24. 10.1093/bioinformatics/btv383 26099265 PMC4595904

[41] Rambaut A, Lam TT, Max Carvalho L, Pybus OG. Exploring the temporal structure of heterochronous sequences using TempEst (formerly Path-O-Gen). Virus Evol. 2016;2(1):vew007 Epub 2016/10/25. 10.1093/ve/vew007 27774300 PMC4989882

[42] Pritchard L, Glover RH, Humphris S, Elphinstone JG, Toth IK. Genomics and taxonomy in diagnostics for food security: soft-rotting enterobacterial plant pathogens. Analytical Methods. 2016;8(1):12–24. 10.1039/c5ay02550h

[43] Stamatakis A. RAxML version 8: a tool for phylogenetic analysis and post-analysis of large phylogenies. Bioinformatics. 2014;30(9):1312–3. Epub 2014/01/24. 10.1093/bioinformatics/btu033 24451623 PMC3998144

[44] Letunic I, Bork P. Interactive tree of life (iTOL) v3: an online tool for the display and annotation of phylogenetic and other trees. Nucleic acids research. 2016;44(W1):W242–5. Epub 2016/04/21. 10.1093/nar/gkw290 27095192 PMC4987883

[45] Huson DH, Bryant D. Application of phylogenetic networks in evolutionary studies. Molecular biology and evolution. 2006;23(2):254–67. Epub 2005/10/14. 10.1093/molbev/msj030 . 16221896

[46] Fischer S, Freuling CM, Muller T, Pfaff F, Bodenhofer U, Hoper D, et al Defining objective clusters for rabies virus sequences using affinity propagation clustering. PLoS Negl Trop Dis. 2018;12(1):e0006182 Epub 2018/01/23. 10.1371/journal.pntd.0006182 29357361 PMC5794188

[47] Bodenhofer U, Kothmeier A, Hochreiter S. APCluster: an R package for affinity propagation clustering. Bioinformatics (Oxford, England). 2011;27(17):2463–4. Epub 2011/07/09. 10.1093/bioinformatics/btr406 . 21737437

[48] Team RC. R: A language and environment for statistical computing. R Foundation for Statistical Computing, Vienna, Austria 2016: htpps://www.R-project.org

[49] Leone M, Sumedha, Weigt M. Clustering by soft-constraint affinity propagation: applications to gene-expression data. Bioinformatics (Oxford, England). 2007;23(20):2708–15. Epub 2007/09/27. 10.1093/bioinformatics/btm414 . 17895277

[50] Frey BJ, Dueck D. Clustering by passing messages between data points. Science (New York, NY). 2007;315(5814):972–6. Epub 2007/01/16. 10.1126/science.1136800 . 17218491

[51] Wong VK, Baker S, Connor TR, Pickard D, Page AJ, Dave J, et al An extended genotyping framework for Salmonella enterica serovar Typhi, the cause of human typhoid. Nature communications. 2016;7:12827 10.1038/ncomms12827 https://www.nature.com/articles/ncomms12827#supplementary-information. PMC5059462 27703135

[52] Schurch AC, Arredondo-Alonso S, Willems RJL, Goering RV. Whole genome sequencing options for bacterial strain typing and epidemiologic analysis based on single nucleotide polymorphism versus gene-by-gene-based approaches. Clinical microbiology and infection: the official publication of the European Society of Clinical Microbiology and Infectious Diseases. 2018;24(4):350–4. Epub 2018/01/09. 10.1016/j.cmi.2017.12.016 . 29309930

[53] Yu J, Blom J, Glaeser SP, Jaenicke S, Juhre T, Rupp O, et al A review of bioinformatics platforms for comparative genomics. Recent developments of the EDGAR 2.0 platform and its utility for taxonomic and phylogenetic studies. Journal of biotechnology. 2017;261:2–9. 10.1016/j.jbiotec.2017.07.010. 28705636

[54] Ekblom R, Wolf JB. A field guide to whole-genome sequencing, assembly and annotation. Evolutionary applications. 2014;7(9):1026–42. Epub 2015/01/02. 10.1111/eva.12178 25553065 PMC4231593

[55] Mahato NK, Gupta V, Singh P, Kumari R, Verma H, Tripathi C, et al Microbial taxonomy in the era of OMICS: application of DNA sequences, computational tools and techniques. Antonie van Leeuwenhoek. 2017;110(10):1357–71. Epub 2017/08/24. 10.1007/s10482-017-0928-1 . 28831610

[56] Loman NJ, Constantinidou C, Chan JZM, Halachev M, Sergeant M, Penn CW, et al High-throughput bacterial genome sequencing: an embarrassment of choice, a world of opportunity. Nature Reviews Microbiology. 2012;10:599 10.1038/nrmicro2850 https://www.nature.com/articles/nrmicro2850#supplementary-information. 22864262

[57] Zielezinski A, Vinga S, Almeida J, Karlowski WM. Alignment-free sequence comparison: benefits, applications, and tools. Genome biology. 2017;18(1):186 10.1186/s13059-017-1319-7 28974235 PMC5627421

[58] Baldauf SL. Phylogeny for the faint of heart: a tutorial. Trends in genetics: TIG. 2003;19(6):345–51. Epub 2003/06/13. 10.1016/s0168-9525(03)00112-4 . 12801728

[59] Brunker K, Marston DA, Horton DL, Cleaveland S, Fooks AR, Kazwala R, et al Elucidating the phylodynamics of endemic rabies virus in eastern Africa using whole-genome sequencing. Virus evolution. 2015;1(1):vev011 Epub 2016/10/25. 10.1093/ve/vev011 27774283 PMC5014479

[60] Karlsson E, Golovliov I, Larkeryd A, Granberg M, Larsson E, Ohrman C, et al Clonality of erythromycin resistance in Francisella tularensis. The Journal of antimicrobial chemotherapy. 2016;71(10):2815–23. Epub 2016/06/24. 10.1093/jac/dkw235 . 27334667

[61] Borile C, Labarre M, Franz S, Sola C, Refregier G. Using affinity propagation for identifying subspecies among clonal organisms: lessons from M. tuberculosis. BMC bioinformatics. 2011;12:224 Epub 2011/06/04. 10.1186/1471-2105-12-224 21635750 PMC3126747

[62] Bi J, Wang Y, Li X, Qi H, Cao H, Xu S. An Adaptive Weighted KNN Positioning Method Based on Omnidirectional Fingerprint Database and Twice Affinity Propagation Clustering. Sensors (Basel, Switzerland). 2018;18(8). Epub 2018/08/04. 10.3390/s18082502 30071642 PMC6111553

[63] Bi T, Li Y, Shekhtman A, Camarero JA. In-cell production of a genetically-encoded library based on the theta-defensin RTD-1 using a bacterial expression system. Bioorganic & medicinal chemistry. 2018;26(6):1212–9. Epub 2017/09/21. 10.1016/j.bmc.2017.09.002 28927803 PMC5840032

[64] Meng J, Zhang J, Luan YS, He XY, Li LS, Zhu YF. Parallel gene selection and dynamic ensemble pruning based on Affinity Propagation. Computers in biology and medicine. 2017;87:8–21. Epub 2017/05/26. 10.1016/j.compbiomed.2017.05.016 . 28544912

[65] Wang J, Chen C, Li HF, Jiang XL, Zhang L. Investigating key genes associated with ovarian cancer by integrating affinity propagation clustering and mutual information network analysis. European review for medical and pharmacological sciences. 2016;20(12):2532–40. Epub 2016/07/08. . 27383302

[66] Prosperi MC, Ciccozzi M, Fanti I, Saladini F, Pecorari M, Borghi V, et al A novel methodology for large-scale phylogeny partition. Nature communications. 2011;2:321 Epub 2011/05/26. 10.1038/ncomms1325 21610724 PMC6045912

[67] Lauber C, Gorbalenya AE. Partitioning the genetic diversity of a virus family: approach and evaluation through a case study of picornaviruses. Journal of virology. 2012;86(7):3890–904. Epub 2012/01/27. 10.1128/jvi.07173-11 22278230 PMC3302503

[68] Maechler Martin, Rousseeuw Peter, Struyf Anja, Hubert Mia, Hornik Kurt, Studer Matthias, et al Finding Groups in Data: Cluster Analysis Extended Rousseeuw, Computes agglomerative hierarchical clustering of the dataset. Repository CRAN. 2016-4-16;(silhouette).

[69] Duchene S, Holt KE, Weill FX, Le Hello S, Hawkey J, Edwards DJ, et al Genome-scale rates of evolutionary change in bacteria. Microb Genom. 2016;2(11):e000094 Epub 2017/03/30. 10.1099/mgen.0.000094 28348834 PMC5320706

[70] Rychener L, InAlbon S, Djordjevic SP, Chowdhury PR, Ziech RE, de Vargas AC, et al Clostridium chauvoei, an Evolutionary Dead-End Pathogen. Front Microbiol. 2017;8:1054 Epub 2017/06/27. 10.3389/fmicb.2017.01054 ; PubMed Central PMCID: PMC5465433. 28649238 PMC5465433

[71] Antwerpen MH, Prior K, Mellmann A, Hoppner S, Splettstoesser WD, Harmsen D. Rapid high resolution genotyping of Francisella tularensis by whole genome sequence comparison of annotated genes ("MLST+"). PloS one. 2015;10(4):e0123298 Epub 2015/04/10. 10.1371/journal.pone.0123298 25856198 PMC4391923

[72] Dempsey MP, Nietfeldt J, Ravel J, Hinrichs S, Crawford R, Benson AK. Paired-end sequence mapping detects extensive genomic rearrangement and translocation during divergence of Francisella tularensis subsp. tularensis and Francisella tularensis subsp. holarctica populations. Journal of bacteriology. 2006;188(16):5904–14. Epub 2006/08/04. 10.1128/jb.00437-06 16885459 PMC1540061

[73] Busch A, Elschner MC, Jacob D, Grunow R, Tomaso H. Draft Genome Sequence of Bacillus anthracis Strain Sterne 09RA8929. Microbiol Resour Announc. 2018;7(14). Epub 2018/12/12. 10.1128/mra.00972-18 30533713 PMC6256644

[74] Kisand V, Lettieri T. Genome sequencing of bacteria: sequencing, de novo assembly and rapid analysis using open source tools. BMC genomics. 2013;14:211 Epub 2013/04/04. 10.1186/1471-2164-14-211 23547799 PMC3618134

