# Supplementary figures and images for: Using affinity propagation clustering for identifying bacterial clades and subclades with whole-genome sequences of Francisella tularensis
Source: PLoS Negl Trop Dis. 2020 Sep 29;14(9):e0008018. doi: 10.1371/journal.pntd.0008018 (PMC7523947; doi:10.1371/journal.pntd.0008018)

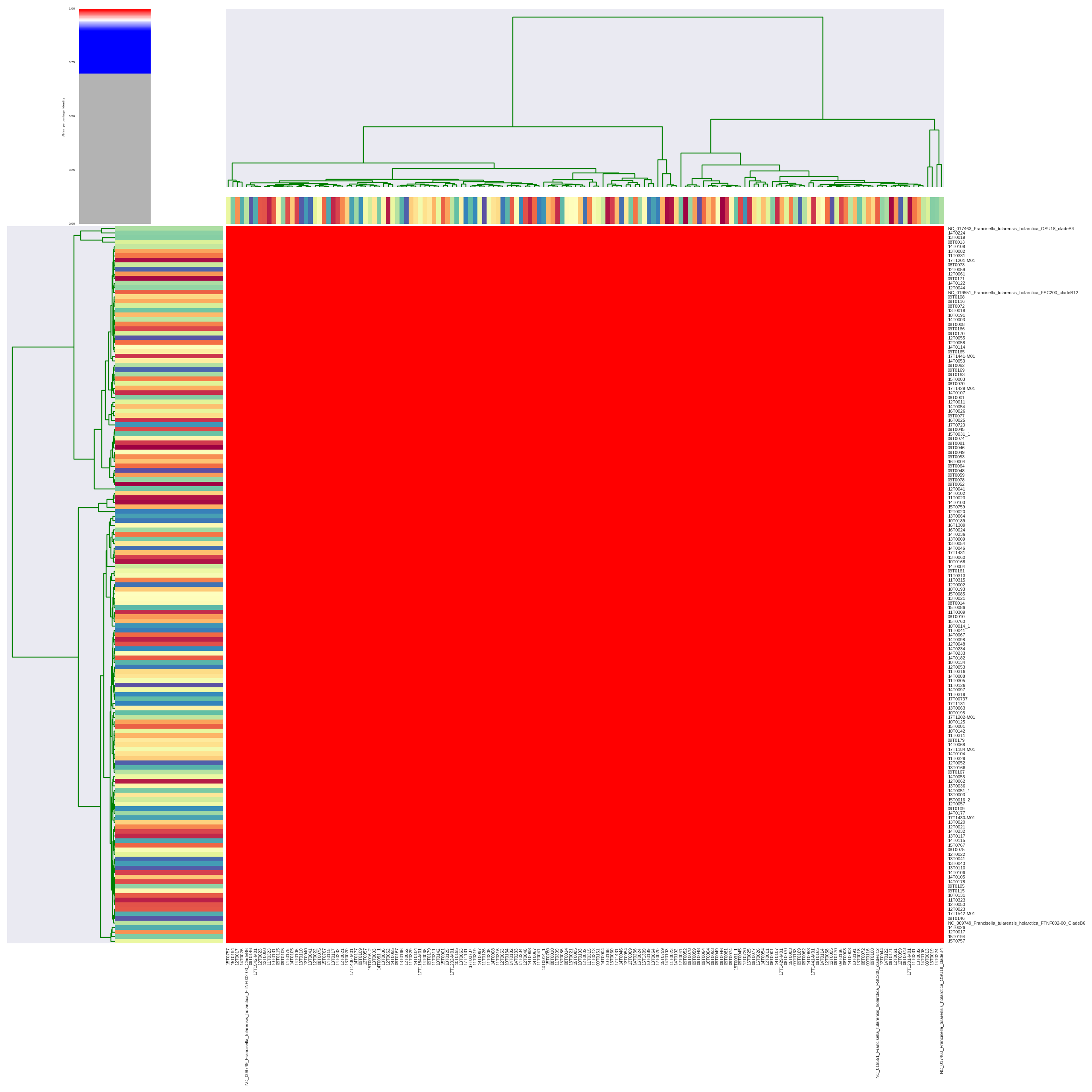

Supplement: S2 Fig — (TIF) [file pntd.0008018.s002.tif]

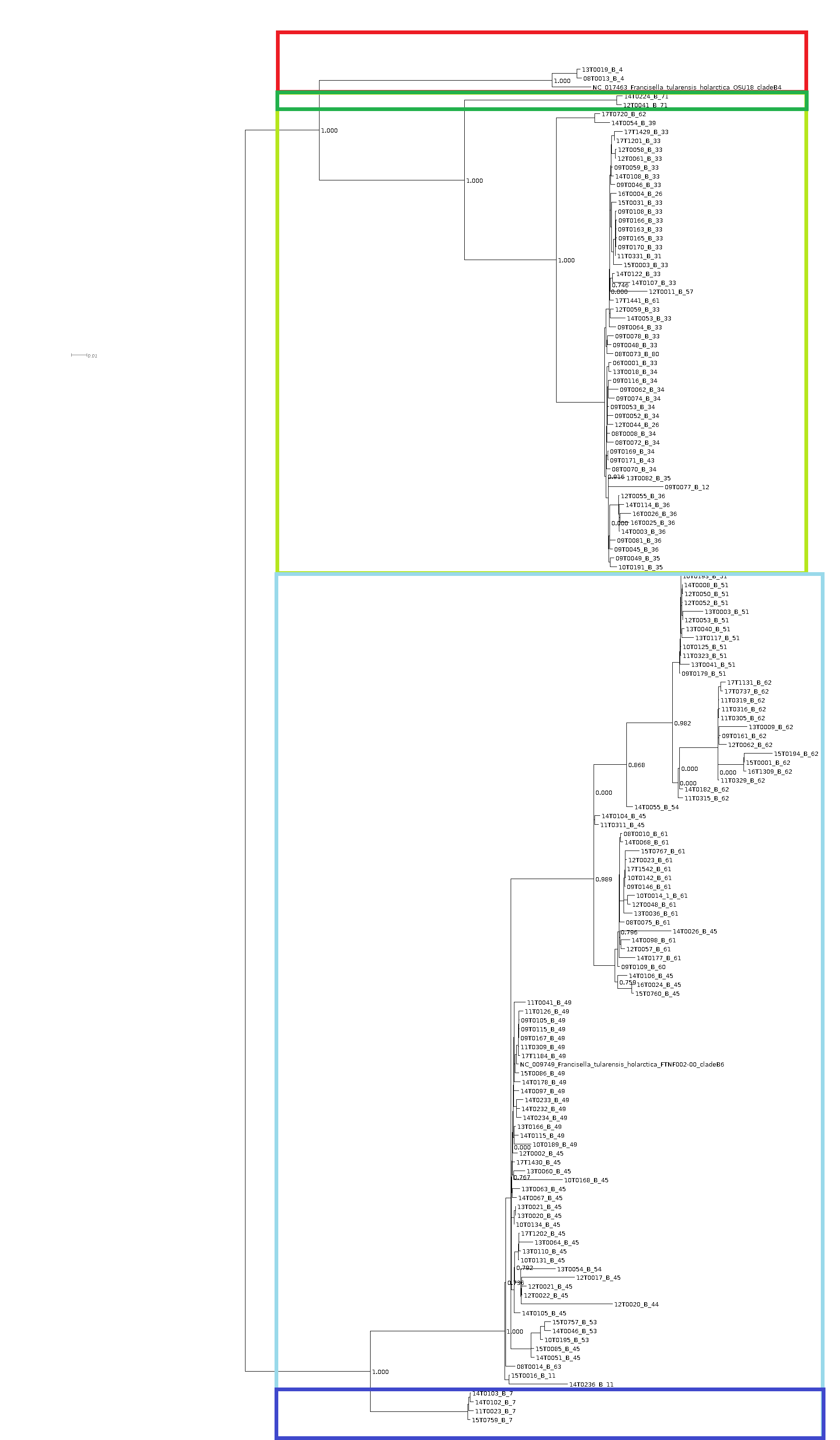

Supplement: S3 Fig — (TIF) [file pntd.0008018.s003.tif]

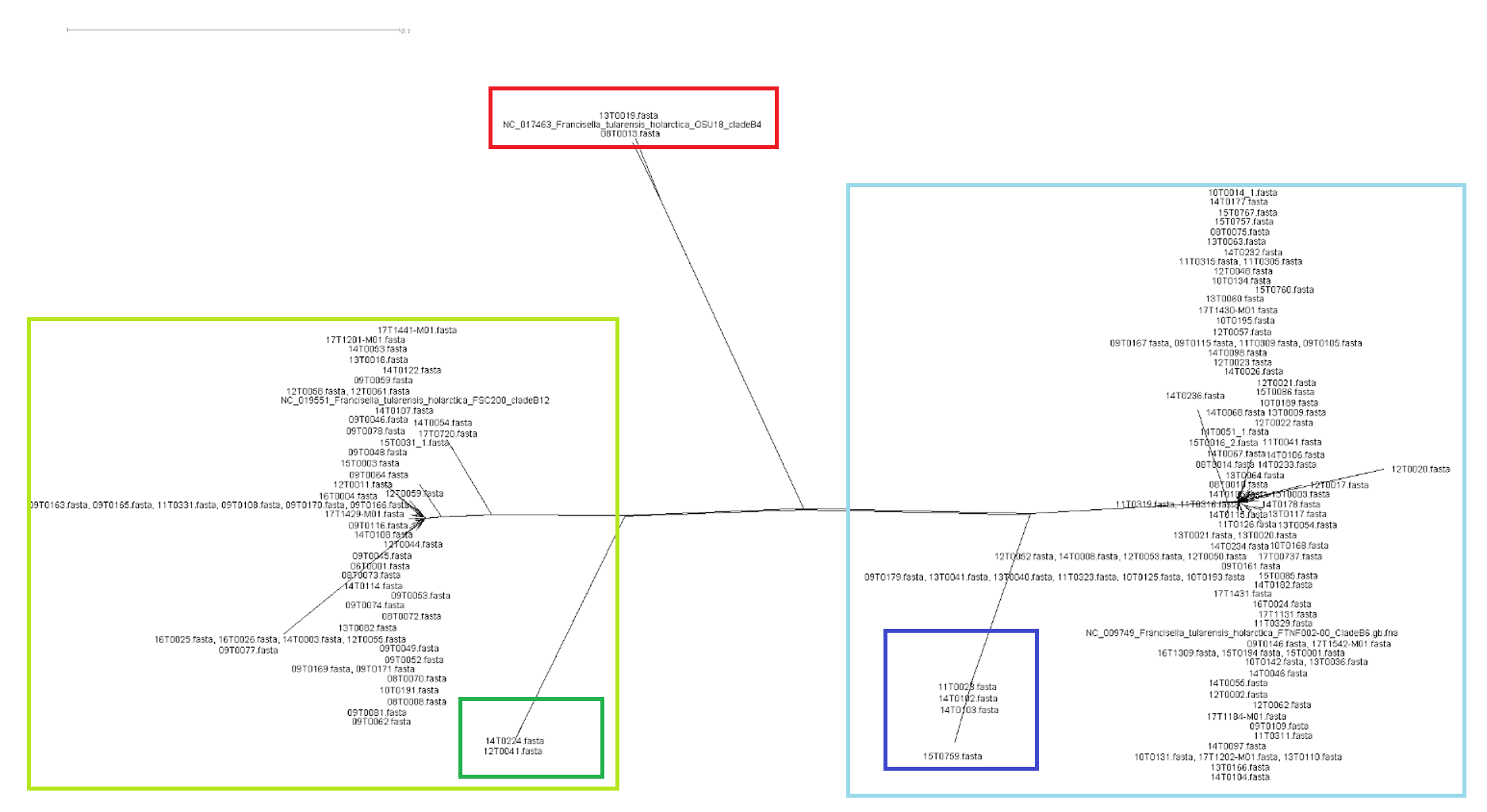

Supplement: S4 Fig — Red lines indicate assignment to clade B.4, green to clade B.12, dark green subclade B.71, blue to clade B.6 and dark blue subclade B.7, canSNPer data included. (TIF) [file pntd.0008018.s004.tif]
