# Supplementary material for: Using affinity propagation clustering for identifying bacterial clades and subclades with whole-genome sequences of Francisella tularensis
Source: PLoS Negl Trop Dis. 2020 Sep 29;14(9):e0008018. doi: 10.1371/journal.pntd.0008018 (PMC7523947; doi:10.1371/journal.pntd.0008018)
Supplement: S1 Table — (x; no result obtained). Isolates hosted by hare and ticks belonging together are defined as follows: 10T0153, 10T0156; 11T0041, 11T0126; 11T0305, 11T0309; 11T0313, 11T0315, 11T0316, 11T0319; 12T0017, 12T0020, 12T0021, 12T0022; 13T0041, 13T0053, 13T0054. (DOCX) [file pntd.0008018.s005.docx]

| FLI-Nr | Clade, PCR verified | place | region | country | host | Subclade with qPCR | canSNPer |
| --- | --- | --- | --- | --- | --- | --- | --- |
| 06T0001 | B.12 | Moorgrund | Thuringia | D | *Lepus europaeus* | B.34 | B.33 |
| 08T0008 | B.12 | Allersberg | Bavaria | D | *Lepus europaeus* | B.13 | B.34 |
| 08T0010 | B.6 | Sehnde | Lower Saxony | D | *Lepus europaeus* | B.18 | B.61 |
| 08T0013 | B4 | Ehingen | Bavaria | D | *Lepus europaeus* | x | B.4 |
| 08T0014 | B.6 | Flacht, Wiessach | Baden-Württemberg | D | *Lepus europaeus* | B.18 | B.63 |
| 08T0070 | B.12 | Kohnsen | Lower Saxony | D | *Lepus europaeus* | B.34 | B.34 |
| 08T0072 | B.12 | Göttingen, Roringen | Lower Saxony | D | *Lepus europaeus* | B.34 | B.34 |
| 08T0073 | B.12 | Rümmer | Lower Saxony | D | *Lepus europaeus* | B.33 | B.80 |
| 08T0075 | B.6 | Würzburg | Bavaria | D | *Lepus europaeus* | B.18 | B.61 |
| 09T0045 | B.12 | AGES in Wien | Lower Austria | AU | *Lepus europaeus* | x | B.36 |
| 09T0046 | B.12 | AGES in Wien | Lower Austria | AU | *Lepus europaeus* | x | B.33 |
| 09T0048 | B.12 | AGES in Wien | Lower Austria | AU | *Lepus europaeus* | x | B.33 |
| 09T0049 | B.12 | Tulln | NÖ | AU | *Lepus europaeus* | x | B.35 |
| 09T0052 | B.12 | AGES in Wien | Lower Austria | AU | *Lepus europaeus* | x | B.34 |
| 09T0053 | B.12 | AGES in Wien | Lower Austria | AU | *Lepus europaeus* | x | B.34 |
| 09T0059 | B.12 | Zwerndorf | NÖ | AU | *Lepus europaeus* | x | B.33 |
| 09T0062 | B.12 | St. Magarethen | Burgenland | AU | *Vulpes vulpes* | x | B.34 |
| 09T0064 | B.12 | Donnerskirchen | B | AU | *Vulpes vulpes* | x | B.33 |
| 09T0074 | B.12 | Zwerndorf | NÖ | AU | *Lepus europaeus* | x | B.34 |
| 09T0077 | B.12 | Grossmugl | Lower Austria | AU | *Lepus europaeus* | x | B.12 |
| 09T0078 | B.12 | Hollabrunn | NÖ | AU | *Lepus europaeus* | x | B.33 |
| 09T0081 | B.12 | Krensdorf | B | AU | *Lepus europaeus* | x | B.36 |
| 09T0105 | B.6 | Geseke | North Rhine-Westphalia | D | *Lepus europaeus* | x | B.49 |
| 09T0108 | B.6 | Geseke | North Rhine-Westphalia | D | *Lepus europaeus* | x | B.33 |
| 09T0109 | B.6 | Markdorf | Baden-Württemberg | D | *Lepus europaeus* | x | B.60 |
| 09T0115 | B.6 | Geseke | North Rhine-Westphalia | D | *Lepus europaeus* | x | B.49 |
| 09T0116 | B.12 | Rabutz | Saxony | D | *Lepus europaeus* | x | B.34 |
| 09T0146 | B.6 | Zeilwald | Baden-Württemberg | D | *Lepus europaeus* | x | B.61 |
| 09T0161 | B.6 | Brake | Lower Saxony | D | *Lepus europaeus* | B.18 | B.62 |
| 09T0163 | B.12 | Wittmund | Lower Saxony | D | *Lepus europaeus* | B.33 | B.33 |
| 09T0165 | B.12 | Wittmund | Lower Saxony | D | *Lepus europaeus* | B.33 | B.33 |
| 09T0166 | B.12 | Wittmund | Lower Saxony | D | *Lepus europaeus* | B.33 | B.33 |
| 09T0167 | B.6 | Wittmund | Lower Saxony | D | *Lepus europaeus* | x | B.49 |
| 09T0169 | B.12 | Wittmund | Lower Saxony | D | *Lepus europaeus* | x | B.34 |
| 09T0170 | B.12 | Wittmund | Lower Saxony | D | *Lepus europaeus* | x | B.33 |
| 09T0171 | B.12 | Wittmund | Lower Saxony | D | *Lepus europaeus* | x | B.43 |
| 09T0179 | B.6 | Geseke | North Rhine-Westphalia | D | *Lepus europaeus* | B.18 | B.51 |
| 10T0014_1 | B.6 | Hemmingen | Baden-Württemberg | D | *Lepus europaeus* | B.18 | B.61 |
| 10T0125 | B.6 | Störmede | North Rhine-Westphalia | D | *Lepus europaeus* | x | B.51 |
| 10T0131 | B.6 | Oppenweiler | Baden-Württemberg | D | *Lepus europaeus* | x | B.45 |
| 10T0134 | B.6 | Ludwigsburg | Baden-Württemberg | D | *Lepus europaeus* | x | B.45 |
| 10T0142 | B.12 | Nordhausen | Thuringia | D | *Homo sapiens sapiens* | x | B.61 |
| 10T0168 | B.6 | Duttenberg | Baden-Württemberg | D | *Lepus europaeus* | x | B.45 |
| 10T0189 | B.6 | Schwanau | Baden-Württemberg | D | *Lepus europaeus* | x | B.49 |
| 10T0191 | B.12 | Stendal | Saxony-Anhalt | D | *Vulpes vulpes* | x | B.35 |
| 10T0193 | B.6 | Geseke | North Rhine-Westphalia | D | *Lepus europaeus* | B.18 | B.51 |
| 10T0195 | B.6 | Lörrach | Baden-Württemberg | D | *Lepus europaeus* | B.18 | B.53 |
| 11T0023 | B.6 | Gießen | Hesse | D | *Vulpes vulpes* | x | B.7 |
| 11T0041 | B.6 | Teningen | Baden-Württemberg | D | *Lepus europaeus* | x | B.49 |
| 11T0126 | B.6 | Soest | North Rhine-Westphalia | D | *Ixodes ricinus* | x | B.49 |
| 11T0305 | B.6 | Soest | North Rhine-Westphalia | D | *Lepus europaeus* | x | B.62 |
| 11T0309 | B.6 | Soest | North Rhine-Westphalia | D | *Ixodes ricinus* | B.18 | B.49 |
| 11T0311 | B.6 | Hadamar | Hesse | D | *Lepus europaeus* | x | B.45 |
| 11T0315 | B.6 | Rüthen | North Rhine-Westphalia | D | *Ixodes ricinus* | B.18 | B.62 |
| 11T0316 | B.6 | Rüthen | North Rhine-Westphalia | D | *Ixodes ricinus* | x | B.62 |
| 11T0319 | B.6 | Rüthen | North Rhine-Westphalia | D | *Ixodes ricinus* | x | B.62 |
| 11T0323 | B.6 | Oesdorf | North Rhine-Westphalia | D | *Lepus europaeus* | x | B51 |
| 11T0329 | B.6 | Störmede | North Rhine-Westphalia | D | *Lepus europaeus* | x | B.62 |
| 11T0331 | B.6 | Störmede | North Rhine-Westphalia | D | *Ixodes ricinus* | B.18 | B.31 |
| 12T0002 | B.6 | Hüls | North Rhine-Westphalia | D | *Lepus europaeus* | B.18 | B.45 |
| 12T0011 | B.12 | Wust | Brandenburg | D | *Castor fiber* | x | B.57 |
| 12T0017 | B.6 | Geseke | North Rhine-Westphalia | D | *Lepus europaeus* | x | B.45 |
| 12T0020 | B.6 | Geseke | North Rhine-Westphalia | D | *Ixodes ricinus* | x | B.44 |
| 12T0021 | B.6 | Geseke | North Rhine-Westphalia | D | *Ixodes ricinus* | x | B.45 |
| 12T0022 | B.6 | Geseke | North Rhine-Westphalia | D | *Ixodes ricinus* | x | B.45 |
| 12T0023 | B.6 | Hochdorf | Baden-Württemberg | D | *Lepus europaeus* | x | B.61 |
| 12T0041 | B.12 | Wust | Brandenburg | D | *Nyctereutes procyonoides* | x | B.71 |
| 12T0044 | B.12 | Wembach | Hesse | D | *Lepus europaeus* | B.34 | B.26 |
| 12T0048 | B.6 | Eberdingen | Baden-Württemberg | D | *Lepus europaeus* | x | B.61 |
| 12T0050 | B.6 | Herringhausen | North Rhine-Westphalia | D | *Lepus europaeus* | x | B.51 |
| 12T0052 | B.6 | Herringhausen | North Rhine-Westphalia | D | *Ixodes ricinus* | x | B.51 |
| 12T0053 | B.6 | Herringhausen | North Rhine-Westphalia | D | *Ixodes ricinus* | x | B.51 |
| 12T0055 | B.12 | Hemmingen | Lower Saxony | D | *Lepus europaeus* | B.36 | B.36 |
| 12T0057 | B.6 | Mainbernheim | Bavaria | D | *Lepus europaeus* | x | B.61 |
| 12T0058 | B.12 | Heideck | Bavaria | D | *Lepus europaeus* | B.33 | B.33 |
| 12T0059 | B.12 | Aurich | Lower Saxony | D | *Lepus europaeus* | B.33 | B.33 |
| 12T0061 | B.12 | Heideck | Bavaria | D | *Lepus europaeus* | B.33 | B.33 |
| 12T0062 | B.6 | Lippstadt | North Rhine-Westphalia | D | *Homo sapiens sapiens* | B.18 | B.62 |
| 13T0003 | B.6 | Lippstadt | North Rhine-Westphalia | D | *Lepus europaeus* | x | B.51 |
| 13T0009 | B.6 | Meiste | North Rhine-Westphalia | D | *Lepus europaeus* | x | B.62 |
| 13T0018 | B.12 | Aspach | Baden-Württemberg | D | *Lepus europaeus* | x | B.34 |
| 13T0019 | B.4 | Leoben | STMK | AU | *Homo sapiens sapiens* | x | B.4 |
| 13T0020 | B.6 | AGES in Wien | Lower Austria | AU | *Ixodes ricinus* | x | B.45 |
| 13T0021 | B.6 | Aspach | Baden-Württemberg | D | *Lepus europaeus* | x | B.45 |
| 13T0036 | B.6 | Aspach | Baden-Württemberg | D | *Lepus europaeus* | x | B.61 |
| 13T0040 | B.6 | Geseke | North Rhine-Westphalia | D | *Lepus europaeus* | x | B.51 |
| 13T0041 | B.6 | Geseke | North Rhine-Westphalia | D | *Lepus europaeus* | x | B.51 |
| 13T0054 | B.6 | Bad Sassendorf | North Rhine-Westphalia | D | *Ixodes ricinus* | x | B.54 |
| 13T0060 | B.6 | Egstedt | Thuringia | D | *Lepus europaeus* | x | B.45 |
| 13T0063 | B.6 | Kernen | Baden-Württemberg | D | *Lepus europaeus* | x | B.45 |
| 13T0064 | B.6 | Aspach | Baden-Württemberg | D | *Lepus europaeus* | x | B.45 |
| 13T0082 | B.12 | Barntrup | North Rhine-Westphalia | D | *Lepus europaeus* | x | B.35 |
| 13T0110 | B.6 | Oppenweiler | Baden-Württemberg | D | *Lepus europaeus* | x | B.45 |
| 13T0117 | B.6 | Holdorf | Lower Saxony | D | *Lepus europaeus* | x | B.51 |
| 13T0166 | B.6 | Geseke | North Rhine-Westphalia | D | *Lepus europaeus* | x | B.49 |
| 14T0003 | B.12 | Hemmingen | Lower Saxony | D | *Lepus europaeus* | x | B.36 |
| 14T0008 | B.6 | Lippstadt | North Rhine-Westphalia | D | *Lepus europaeus* | x | B.51 |
| 14T0026 | B.6 | Oberriexingen | Baden-Württemberg | D | *Lepus europaeus* | x | B.45 |
| 14T0046 | B.6 | Pellheim | Bavaria | D | *Lepus europaeus* | x | B.53 |
| 14T0051 | B.6 | Erding | Bavaria | D | *Lepus europaeus* | x | B.45 |
| 14T0053 | B.12 | Erding | Bavaria | D | *Lepus europaeus* | x | B.33 |
| 14T0054 | B.12 | Puch | Bavaria | D | *Lepus europaeus* | x | B.39 |
| 14T0055 | B.6 | Seulbitz | Bavaria | D | *Lepus europaeus* | x | B.54 |
| 14T0067 | B.6 | Holdorf | Lower Saxony | D | *Lepus europaeus* | x | B.45 |
| 14T0068 | B.6 | Sehnde | Lower Saxony | D | *Lepus europaeus* | x | B.61 |
| 14T0097 | B.6 | Dettelbach | Bavaria | D | *Lepus europaeus* | x | B.49 |
| 14T0098 | B.6 | Bad Säckingen | Baden-Württemberg | D | *Homo sapiens sapiens* | x | B.61 |
| 14T0102 | B.6 | Bad Münder | Lower Saxony | D | *Glis glis* | x | B.7 |
| 14T0103 | B.6 | Bad Münder | Lower Saxony | D | *Glis glis* | x | B.7 |
| 14T0104 | B.6 | Hilter | Lower Saxony | D | *Lepus europaeus* | x | B.45 |
| 14T0105 | B.6 | Kürnach | Bavaria | D | *Lepus europaeus* | x | B.45 |
| 14T0106 | B.6 | Werneck | Bavaria | D | *Lepus europaeus* | x | B.45 |
| 14T0107 | B.12 | Zörbitz | Saxony-Anhalt | D | *Lepus europaeus* | x | B.33 |
| 14T0108 | B.12 | Dörna | Thuringia | D | *Lepus europaeus* | x | B.33 |
| 14T0114 | B.12 | Hemmingen | Lower Saxony | D | *Lepus europaeus* | x | B.36 |
| 14T0115 | B.6 | Chemnitz | Saxony | D | *Homo sapiens sapiens* | x | B.49 |
| 14T0122 | B.12 | Erding | Bavaria | D | *Lepus europaeus* | x | B.33 |
| 14T0177 | B.6 | Ebern | Bavaria | D | *Lepus europaeus* | x | B.61 |
| 14T0178 | B.6 | Mayen | Rhineland-Palatinate | D | *Lepus europaeus* | x | B.49 |
| 14T0182 | B.6 | Lippe | North Rhine-Westphalia | D | *Lepus europaeus* | x | B.62 |
| 14T0224 | B.12 | Rickert | Schleswig-Holstein | D | *Lepus europaeus* | x | B.71 |
| 14T0232 | B.6 | Grettstadt | Bavaria | D | *Lepus europaeus* | x | B.49 |
| 14T0233 | B.6 | Grettstadt | Bavaria | D | *Lepus europaeus* | x | B.49 |
| 14T0234 | B.6 | Grettstadt | Bavaria | D | *Lepus europaeus* | x | B.49 |
| 14T0236 | B.6 | Lippe | North Rhine-Westphalia | D | *Lepus europaeus* | x | B.11 |
| 15T0001 | B.6 | Ehingen | Bavaria | D | *Lepus europaeus* | x | B.62 |
| 15T0003 | B.12 | Bad Neustadt | Bavaria | D | *Lepus europaeus* | x | B33 |
| 15T0016 | B.6 | Lippe | North Rhine-Westphalia | D | *Lepus europaeus* | B.18 | B.11 |
| 15T0031 | B.12 | x | North Rhine-Westphalia | D | *Lepus europaeus* | B.34 | B.33 |
| 15T0085 | B.6 | Euskirchen | North Rhine-Westphalia | D | *Lepus europaeus* | B.18 | B.45 |
| 15T0086 | B.6 | Euskirchen | North Rhine-Westphalia | D | *Lepus europaeus* | B.18 | B.49 |
| 15T0194 | B.6 | Bechhofen | Bavaria | D | *Lepus europaeus* | x | B.62 |
| 15T0757 | x | Dachau | Bavaria | D | *Lepus europaeus* | x | B.53 |
| 15T0759 | x | Goslar | Lower Saxony | D | *Lepus europaeus* | x | B.7 |
| 15T0760 | x | Bad Kreuznach | Rhineland-Palatinate | D | *Lepus europaeus* | x | B.45 |
| 15T0767 | B.6 | Ludwigsburg | Baden-Württemberg | D | *Lepus europaeus* | x | B.61 |
| 16T0004 | x | Cham | Bavaria | D | *Lepus europaeus* | x | B.26 |
| 16T0024 | x | Rüdersheim | Rhineland-Palatinate | D | *Lepus europaeus* | B.18 | B.45 |
| 16T0025 | x | Hannover | Lower Saxony | D | *Lepus europaeus* | x | B.36 |
| 16T0026 | x | Hemmingen | Lower Saxony | D | *Lepus europaeus* | x | B.36 |
| 16T1309 | x | x | Lower Saxony | D | *Lepus europaeus* | x | B.62 |
| 17T0720 | x | Xanten | North Rhine-Westphalia | D | *Lepus europaeus* | x | B.62 |
| 17T0737 | x | Kalkstein bei Ahlen | North Rhine-Westphalia | D | *Lepus europaeus* | x | B.62 |
| 17T1131 | x | Lippetal | North Rhine-Westphalia | D | *Lepus europaeus* | x | B.62 |
| 17T1184 | x | Gesecke | North Rhine-Westphalia | D | *Lepus europaeus* | x | B.49 |
| 17T1201 | x | Wertingen | Bavaria | D | *Lepus europaeus* | x | B.33 |
| 17T1202 | x | Kirchberg/Murr | Baden-Württemberg | D | *Lepus europaeus* | x | B.45 |
| 17T1429 | x | Laugna | Bavaria | D | *Lepus europaeus* | x | B.33 |
| 17T1430 | x | Miesbach | Bavaria | D | *Lepus europaeus* | x | B.45 |
| 17T1441 | x | x | x | x | *x* | x | B.61 |
| 17T1542 | x | Backnang | Baden-Württemberg | D | *Lepus europaeus* | x | B.61 |
| NC_009749_Francisella_tularensis_holarctica_FTNF002-00_CladeB6 | B.6 | x | x | x | x | x | x |
| NC_017463_Francisella_tularensis_holarctica_OSU18_cladeB4 | B.4 | x | x | x | x | x | x |
| NC_019551_Francisella_tularensis_holarctica_FSC200_cladeB12 | B.12 | x | x | x | x | x | x |
