# Supplementary material for: Using affinity propagation clustering for identifying bacterial clades and subclades with whole-genome sequences of Francisella tularensis
Source: PLoS Negl Trop Dis. 2020 Sep 29;14(9):e0008018. doi: 10.1371/journal.pntd.0008018 (PMC7523947; doi:10.1371/journal.pntd.0008018)
Supplement: S2 Table — (DOCX) [file pntd.0008018.s006.docx]

|  | Number of Sequences | Number of SNPs |
| --- | --- | --- |
| Variations for all sequences | 155 | 1,517 |
| Cluster B.6 | 92 | 371 |
| Cluster B.12 | 53 | 358 |
| Cluster B.4 | 3 | 65 |
| Cluster B.7 (subclade to B.6) | 4 | 37 |
| Cluster B.71 (subclade to B.12) | 2 | 9 |
