# Supplementary material for: Using affinity propagation clustering for identifying bacterial clades and subclades with whole-genome sequences of Francisella tularensis
Source: PLoS Negl Trop Dis. 2020 Sep 29;14(9):e0008018. doi: 10.1371/journal.pntd.0008018 (PMC7523947; doi:10.1371/journal.pntd.0008018)
Supplement: S3 Table — (DOCX) [file pntd.0008018.s007.docx]

| Name | APC, based on ANI | APC, based on parSNP | APC, based on kSNP | HierBAPS, | HierBAPS, |
| --- | --- | --- | --- | --- | --- |
|  |  |  |  | Clustering 1 | Clustering 2 |
| 06T0001 | 2 | 2 | 2 | 2 | 3 |
| 08T0008 | 2 | 2 | 2 | 2 | 3 |
| 08T0010 | 1 | 1 | 1 | 1 | 1 |
| 08T0013 | 3 | 3 | 3 | 4 | 8 |
| 08T0014 | 1 | 1 | 1 | 1 | 1 |
| 08T0070 | 2 | 2 | 2 | 2 | 3 |
| 08T0072 | 2 | 2 | 2 | 2 | 3 |
| 08T0073 | 2 | 2 | 2 | 2 | 3 |
| 08T0075 | 1 | 1 | 1 | 1 | 1 |
| 09T0045 | 2 | 2 | 2 | 2 | 3 |
| 09T0046 | 2 | 2 | 2 | 2 | 3 |
| 09T0048 | 2 | 2 | 2 | 2 | 3 |
| 09T0049 | 2 | 2 | 2 | 2 | 3 |
| 09T0052 | 2 | 2 | 2 | 2 | 3 |
| 09T0053 | 2 | 2 | 2 | 2 | 3 |
| 09T0059 | 2 | 2 | 2 | 2 | 3 |
| 09T0062 | 2 | 2 | 2 | 2 | 3 |
| 09T0064 | 2 | 2 | 2 | 2 | 3 |
| 09T0074 | 2 | 2 | 2 | 2 | 3 |
| 09T0077 | 2 | 2 | 2 | 2 | 5 |
| 09T0078 | 2 | 2 | 2 | 2 | 3 |
| 09T0081 | 2 | 2 | 2 | 2 | 3 |
| 09T0105 | 1 | 1 | 1 | 1 | 1 |
| 09T0108 | 2 | 2 | 2 | 2 | 3 |
| 09T0109 | 1 | 1 | 1 | 1 | 1 |
| 09T0115 | 1 | 1 | 1 | 1 | 1 |
| 09T0116 | 2 | 2 | 2 | 2 | 3 |
| 09T0146 | 1 | 1 | 1 | 1 | 1 |
| 09T0161 | 1 | 1 | 1 | 1 | 1 |
| 09T0163 | 2 | 2 | 2 | 2 | 3 |
| 09T0165 | 2 | 2 | 2 | 2 | 3 |
| 09T0166 | 2 | 2 | 2 | 2 | 3 |
| 09T0167 | 1 | 1 | 1 | 1 | 1 |
| 09T0169 | 2 | 2 | 2 | 2 | 3 |
| 09T0170 | 2 | 2 | 2 | 2 | 3 |
| 09T0171 | 2 | 2 | 2 | 2 | 3 |
| 09T0179 | 1 | 1 | 1 | 1 | 1 |
| 10T0014 | 1 | 1 | 1 | 1 | 1 |
| 10T0125 | 1 | 1 | 1 | 1 | 1 |
| 10T0131 | 1 | 1 | 1 | 1 | 1 |
| 10T0134 | 1 | 1 | 1 | 1 | 1 |
| 10T0142 | 1 | 1 | 1 | 1 | 1 |
| 10T0168 | 1 | 1 | 1 | 1 | 1 |
| 10T0189 | 1 | 1 | 1 | 1 | 1 |
| 10T0191 | 2 | 2 | 2 | 2 | 3 |
| 10T0193 | 1 | 1 | 1 | 1 | 1 |
| 10T0195 | 1 | 1 | 1 | 1 | 1 |
| 11T0023 | 1 | 1 | 1 | 3 | 7 |
| 11T0041 | 1 | 1 | 1 | 1 | 1 |
| 11T0126 | 1 | 1 | 1 | 1 | 1 |
| 11T0305 | 1 | 1 | 1 | 1 | 1 |
| 11T0309 | 1 | 1 | 1 | 1 | 1 |
| 11T0311 | 1 | 1 | 1 | 1 | 1 |
| 11T0315 | 1 | 1 | 1 | 1 | 1 |
| 11T0316 | 1 | 1 | 1 | 1 | 1 |
| 11T0319 | 1 | 1 | 1 | 1 | 1 |
| 11T0323 | 1 | 1 | 1 | 1 | 1 |
| 11T0329 | 1 | 1 | 1 | 1 | 1 |
| 11T0331 | 2 | 2 | 2 | 2 | 3 |
| 12T0002 | 1 | 1 | 1 | 1 | 1 |
| 12T0011 | 2 | 2 | 2 | 2 | 3 |
| 12T0017 | 1 | 1 | 1 | 1 | 1 |
| 12T0020 | 1 | 1 | 1 | 1 | 2 |
| 12T0021 | 1 | 1 | 1 | 1 | 1 |
| 12T0022 | 1 | 1 | 1 | 1 | 1 |
| 12T0023 | 1 | 1 | 1 | 1 | 1 |
| 12T0041 | 2 | 2 | 2 | 4 | 9 |
| 12T0044 | 2 | 2 | 2 | 2 | 3 |
| 12T0048 | 1 | 1 | 1 | 1 | 1 |
| 12T0050 | 1 | 1 | 1 | 1 | 1 |
| 12T0052 | 1 | 1 | 1 | 1 | 1 |
| 12T0053 | 1 | 1 | 1 | 1 | 1 |
| 12T0055 | 2 | 2 | 2 | 2 | 3 |
| 12T0057 | 1 | 1 | 1 | 1 | 1 |
| 12T0058 | 2 | 2 | 2 | 2 | 3 |
| 12T0059 | 2 | 2 | 2 | 2 | 3 |
| 12T0061 | 2 | 2 | 2 | 2 | 3 |
| 12T0062 | 1 | 1 | 1 | 1 | 1 |
| 13T0003 | 1 | 1 | 1 | 1 | 1 |
| 13T0009 | 1 | 1 | 1 | 1 | 1 |
| 13T0018 | 2 | 2 | 2 | 2 | 3 |
| 13T0019 | 3 | 3 | 3 | 4 | 8 |
| 13T0020 | 1 | 1 | 1 | 1 | 1 |
| 13T0021 | 1 | 1 | 1 | 1 | 1 |
| 13T0036 | 1 | 1 | 1 | 1 | 1 |
| 13T0040 | 1 | 1 | 1 | 1 | 1 |
| 13T0041 | 1 | 1 | 1 | 1 | 1 |
| 13T0054 | 1 | 1 | 1 | 1 | 1 |
| 13T0060 | 1 | 1 | 1 | 1 | 1 |
| 13T0063 | 1 | 1 | 1 | 1 | 1 |
| 13T0064 | 1 | 1 | 1 | 1 | 1 |
| 13T0082 | 2 | 2 | 2 | 2 | 3 |
| 13T0110 | 1 | 1 | 1 | 1 | 1 |
| 13T0117 | 1 | 1 | 1 | 1 | 1 |
| 13T0166 | 1 | 1 | 1 | 1 | 1 |
| 14T0003 | 2 | 2 | 2 | 2 | 3 |
| 14T0008 | 1 | 1 | 1 | 1 | 1 |
| 14T0026 | 1 | 1 | 1 | 1 | 1 |
| 14T0046 | 1 | 1 | 1 | 1 | 1 |
| 14T0051 | 1 | 1 | 1 | 1 | 1 |
| 14T0053 | 2 | 2 | 2 | 2 | 3 |
| 14T0054 | 2 | 2 | 2 | 2 | 4 |
| 14T0055 | 1 | 1 | 1 | 1 | 1 |
| 14T0067 | 1 | 1 | 1 | 1 | 1 |
| 14T0068 | 1 | 1 | 1 | 1 | 1 |
| 14T0097 | 1 | 1 | 1 | 1 | 1 |
| 14T0098 | 1 | 1 | 1 | 1 | 1 |
| 14T0102 | 1 | 1 | 1 | 3 | 7 |
| 14T0103 | 1 | 1 | 1 | 3 | 7 |
| 14T0104 | 1 | 1 | 1 | 1 | 1 |
| 14T0105 | 1 | 1 | 1 | 1 | 1 |
| 14T0106 | 1 | 1 | 1 | 1 | 1 |
| 14T0107 | 2 | 2 | 2 | 2 | 3 |
| 14T0108 | 2 | 2 | 2 | 2 | 3 |
| 14T0114 | 2 | 2 | 2 | 2 | 3 |
| 14T0115 | 1 | 1 | 1 | 1 | 1 |
| 14T0122 | 2 | 2 | 2 | 2 | 3 |
| 14T0177 | 1 | 1 | 1 | 1 | 1 |
| 14T0178 | 1 | 1 | 1 | 1 | 1 |
| 14T0182 | 1 | 1 | 1 | 1 | 1 |
| 14T0224 | 2 | 2 | 2 | 4 | 9 |
| 14T0232 | 1 | 1 | 1 | 1 | 1 |
| 14T0233 | 1 | 1 | 1 | 1 | 1 |
| 14T0234 | 1 | 1 | 1 | 1 | 1 |
| 14T0236 | 1 | 1 | 1 | 1 | 2 |
| 15T0001 | 1 | 1 | 1 | 1 | 1 |
| 15T0003 | 2 | 2 | 2 | 2 | 3 |
| 15T0016 | 1 | 1 | 1 | 1 | 1 |
| 15T0031 | 2 | 2 | 2 | 2 | 3 |
| 15T0085 | 1 | 1 | 1 | 1 | 1 |
| 15T0086 | 1 | 1 | 1 | 1 | 1 |
| 15T0194 | 1 | 1 | 1 | 1 | 1 |
| 15T0757 | 1 | 1 | 1 | 1 | 1 |
| 15T0759 | 1 | 1 | 1 | 3 | 6 |
| 15T0760 | 1 | 1 | 1 | 1 | 1 |
| 15T0767 | 1 | 1 | 1 | 1 | 1 |
| 16T0004 | 2 | 2 | 2 | 2 | 3 |
| 16T0024 | 1 | 1 | 1 | 1 | 1 |
| 16T0025 | 2 | 2 | 2 | 2 | 3 |
| 16T0026 | 2 | 2 | 2 | 2 | 3 |
| 16T1309 | 1 | 1 | 1 | 1 | 1 |
| 17T00737 | 1 | 1 | 1 | 1 | 1 |
| 17T0720 | 2 | 2 | 2 | 2 | 4 |
| 17T1131 | 1 | 1 | 1 | 1 | 1 |
| 17T1184 | 1 | 1 | 1 | 1 | 1 |
| 17T1201 | 2 | 2 | 2 | 2 | 3 |
| 17T1202 | 1 | 1 | 1 | 1 | 1 |
| 17T1429 | 2 | 2 | 2 | 2 | 3 |
| 17T1430 | 1 | 1 | 1 | 1 | 1 |
| 17T1431 | 1 | 1 | 1 | 1 | 1 |
| 17T1441- | 2 | 2 | 2 | 2 | 3 |
| 17T1542 | 1 | 1 | 1 | 1 | 1 |
| NC_009749_Francisella_tularensis_holarctica_FTNF002-00_CladeB6 | 1 | 1 | 1 | 1 | 1 |
| NC_017463_Francisella_tularensis_holarctica_OSU18_cladeB4 | 3 | 3 | 3 | 4 | 8 |
| NC_019551_Francisella_tularensis_holarctica_FSC200_cladeB12 | 2 | 3 | 2 | 2 | 3 |
