# Supplementary material for: Using affinity propagation clustering for identifying bacterial clades and subclades with whole-genome sequences of Francisella tularensis
Source: PLoS Negl Trop Dis. 2020 Sep 29;14(9):e0008018. doi: 10.1371/journal.pntd.0008018 (PMC7523947; doi:10.1371/journal.pntd.0008018)
Supplement: S4 Table — (DOCX) [file pntd.0008018.s008.docx]

| ANI | ANI | ksnp | ksnp | parsnp | parsnp |
| --- | --- | --- | --- | --- | --- |
| possible plateaus | proportional distribution [%] | possible plateaus | proportional distribution [%] | possible plateaus | proportional distribution [%] |
| **3** | **76,2** | **3** | **34,3** | **3** | **63,6** |
| 4 | 8,9 | 4 | 20,0 | 4 | 13,6 |
| 5 | 8,4 | 5 | 8,6 | 5 | 9,1 |
| 6 | 2,0 | 7 | 5,7 | 6 | 9,1 |
| 7 | 1,6 | 8 | 2,9 | 132 | 4,5 |
| 8 | 0,5 | 10 | 2,9 |  |  |
| 9 | 0,3 | 13 | 2,9 |  |  |
| 10 | 0,4 | 17 | 2,9 |  |  |
| 11 | 0,2 | 36 | 2,9 |  |  |
| 13 | 0,2 | 154 | 2,9 |  |  |
| 14 | 0,2 | 155 | 14,3 |  |  |
| 15 | 0,1 |  |  |  |  |
| 17 | 0,1 |  |  |  |  |
| 18 | 0,1 |  |  |  |  |
| 19 | 0,1 |  |  |  |  |
| 20 | 0,1 |  |  |  |  |
| 21 | 0,1 |  |  |  |  |
| 23 | 0,1 |  |  |  |  |
| 25 | 0,1 |  |  |  |  |
| 26 | 0,1 |  |  |  |  |
| 28 | 0,1 |  |  |  |  |
| 34 | 0,1 |  |  |  |  |
| 35 | 0,1 |  |  |  |  |
| 38 | 0,1 |  |  |  |  |
| 42 | 0,1 |  |  |  |  |
| 50 | 0,1 |  |  |  |  |
| 61 | 0,1 |  |  |  |  |
| 85 | 0,1 |  |  |  |  |
| 115 | 0,1 |  |  |  |  |
| 155 | 0,1 |  |  |  |  |
